# Imidazolium-labeled glycosides as probes to harness glycosyltransferase activity in human breast milk†
†Electronic supplementary information (ESI) available: Full experimental and characterization data for all compounds, including NMR spectra and LC-MS traces. See DOI: 10.1039/c7ob00550d


**DOI:** 10.1039/c7ob00550d

**Published:** 2017-04-06

**Authors:** I. Sittel, M. C. Galan

**Affiliations:** a School of Chemistry , University of Bristol , Cantock's Close , Bristol BS8 1TS , UK . Email: m.c.galan@bristol.ac.uk ; Fax: (+)44 (0)117 925 1295

## Abstract

Imidazolium-labeled (ITag-) glycosides are used to harness the glycosyltransferase activity directly from human breast milk. The covalently attached ionic labels provide a bifunctional chemical handle that is used to monitor reaction progress by MS, as well as aid in product purification from complex mixtures. The technology is exemplified in the synthesis of biologically relevant oligosaccharide analogs, LacNAc-ITag, ITag-Lewis^x^ and ITag-Lewis^a^, in a matter of days from human breast milk without having to isolate specific enzymes.

## Abstract 2

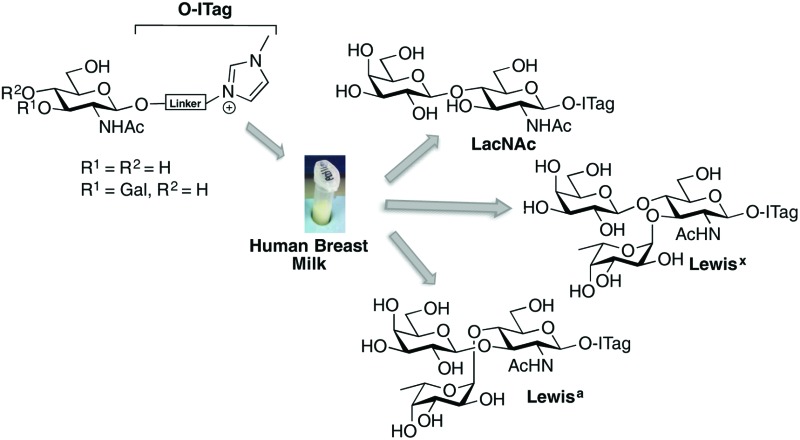
Imidazolium-labeled (ITag-) glycosides have been used to harness the glycosyltransferase activity directly from human breast milk (HBM). The technology is exemplified in the synthesis of biologically relevant oligosaccharide analogs, ITag-LacNAc, ITag-Lewis^x^ and ITag-Lewis^a^, in a matter of days from (HBM) without isolating the enzymes.

## 

The growing appreciation of oligosaccharides and glycoconjugates as key mediators in many important biological processes from cell signaling, embryogenesis, neural development to pathogen recognition, inflammation, innate immune responses and cancer, has led to their use as probes for biological research and as key compounds for drug and vaccine development.^1^ Despite the many recent advances in the field, progress in this area is still impeded by a lack of general and efficient methods for the routine preparation of complex oligosaccharides. Traditional chemical approaches rely on lengthy and time consuming chemical steps to achieve good yields and stereocontrol.^2^ On the other hand, the use of carbohydrate processing enzymes such as glycosyltransferases, to construct oligosaccharides offers several advantages over chemical approaches, since these enzymes are able to form glycosidic bonds with exquisite regio- and stereocontrol by catalyzing the transfer of an activated sugar donor to growing oligosaccharide chains without the need for protecting groups.2d,^3^ However, access to these enzymes is limited as the proteins can be challenging to express, expensive to employ on scale and only a handful are currently commercially available.^4^

Human breast milk (HBM) is an extremely complex and highly variable biofluid comprised of carbohydrates, proteins, lipids and enzymes, which has evolved over millennia to nourish infants and protect them from disease, whilst their own immune system matures.^5^ HBM contains more than 200 different unbound human milk oligosaccharides (HMOs),5b,^6^ consisting of five monosaccharide building blocks: galactose (Gal), glucose (Glc), *N*-acetylglucosamine (GlcNAc), fucose (Fuc) and sialic acid (Neu5Ac). All HMOs carry lactose (Gal-β-1,4-Glc) at the reducing end, which can be elongated through a β-1,3- or β-1,6-linkage by two different disaccharides, either Gal-β-1,3-GlcNAc (lacto-*N*-biose, type 1 chain) or Gal-β-1,4-GlcNAc *N*-acetyllactosamine (LacNAc, type 2 chain). Further chain elongation can take place by the incorporation of Fuc and Neu5Ac units.^7^ Our understanding of the biosynthesis of these oligosaccharides is limited, since most of the specific glycosyltransferases that are responsible for the formation of HMO structures with specific glycosidic linkages have not been identified.7b,^8^ In addition, HMO-specific glycosyltransferase expression levels are determined by the blood group, diet and environmental and genetic factors as well as lactational period of the donor.7a,^9^

Ionic liquid-based labels have emerged as a new class of chemical labels that can be employed for the immobilization and mass spectrometry (MS) detection of reagents from complex reaction mixtures, due to their unique physical and chemical properties.^10^ Our group10c,^11^ and others^12^ have reported the synthesis of inexpensive and versatile imidazolium-based tags (ITags) and their application to chromatography-free chemical oligosaccharide synthesis. Furthermore, in our work, we demonstrated that the cationic labels are also compatible with enzymatic transformations and that ITag-glycoside substrates could be employed for the qualitative and quantitative reaction monitoring, using MS techniques, of bovine milk β-1,4-galactosyltransferase (β-1,4-GalT),^13^ human α-1,3-fucosyltransferase (α-1,3-FucT VI)^14^ and α-2,3-sialyltransferase (α-2,3-ST) from *pasteurella multocida*, as well as α-2,6-sialyltransferase (α-2,6-ST)^15^ from *photobacterium damsela*. More recently, ITag-labeled mannosamine was also employed for the non-covalent labeling of live cells,^16^ demonstrating the versatility of the probes.

Encouraged by the simplicity and practicality of the approach, we hypothesized that ITag-glycan probes would be ideally suited to harness the biosynthetic machinery (*e.g.* glycosyltransferases) present in HBM, and thus gain expedient access to biologically important oligosaccharides, without the need to isolate or express specific enzymes (Fig. 1).

**Fig. 1:**
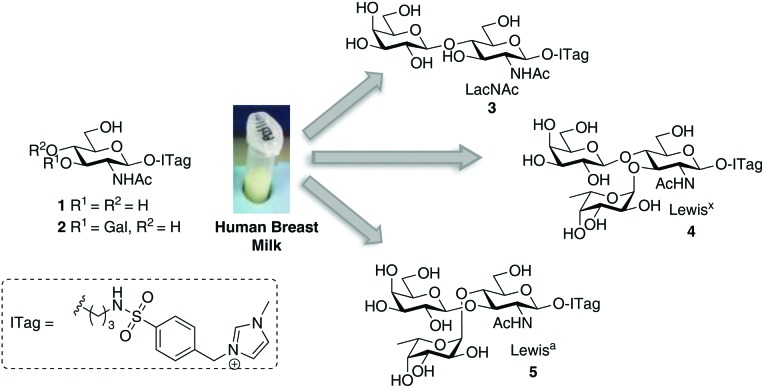
General strategy to harness glycosyltransferase activity in human breast milk.

Initial experiments began by assessing our ability to monitor mixtures of ITag-glycoside products that had been generated *in vitro* in a multi-enzyme environment. To that end, methylimidazolium *N*-benzenesulfonyl-linked GlcNAc (GlcNAc-ITag) **1**, which was prepared in 4 steps and 59% overall yield from azidopropyl GlcNAc **6** 15 (see ESI† for details), was incubated in 3-morpholinopropane-1-sulfonic acid (MOPS) buffer (pH 7.8) at 37 °C with three different recombinant enzymes: β-1,4-GalT, which catalyzes the transfer of galactose from uridine 5′-diphosphogalactose (UDP-Gal) to C4 of GlcNAc to yield ITag-LacNAc **3**; α-2,3-ST, which in the presence of cytidine 5′-monophospho-*N*-acetylneuraminic acid (CMP-Neu5Ac) will add Neu5Ac at C3′ of the LacNAc disaccharide to yield 3′-Sialyl-LacNAc-ITag **9** and α-1,3-FucT VI, which can fucosylate **3** at C3 of the GlcNAc unit in the presence of guanosine 5′-diphospho-β-l-fucose (GDP-Fuc) to form the ITag-Lewis^X^ trisaccharide [Fuc-α-1,3-(Gal-β-1,4-)GlcNAc] **4** or generate Sialyl-Lewis^X^-ITag **10** from **9** 17 (Scheme 1). Incubations were carried out in the presence of an excess of an equimolar mixture of UDP-Gal, CMP-Neu5Ac and GDP-Fuc, which was added portion-wise over 3 days. LC-MS was used to analyze and monitor reaction progress. Four different ITagged species were identified in the mixture: starting material **1** ([M^+^] 513), LacNAc-ITag **3** ([M^+^] 675) and trisaccharides **4** ([M^+^] 821) and **9** ([M^+^] 966) in a ratio of 12 : 42 : 2 : 44 for **1** : **3** : **4** : **9** as determined by LC-MS. Comparison of retention times and MS data to that of isolated ITag-materials,^13^–^15^ confirmed the structure identity of the individual ITag-glycosides.

**Scheme 1:**
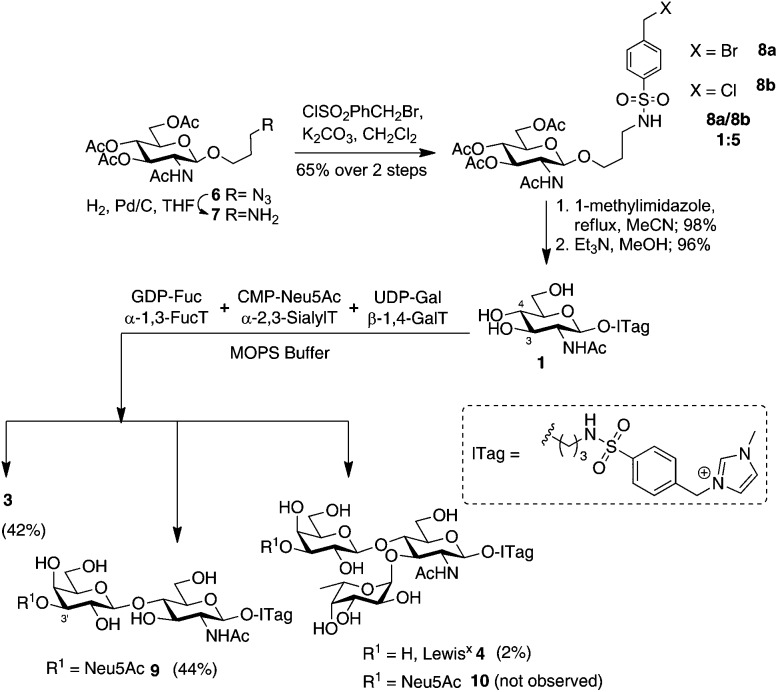
One-pot *in vitro* chemo-enzymatic synthesis of ITagged human milk oligosaccharides **3**, **4** and **9**. See ESI Fig. S1† for traces.

Having demonstrated that we are able to detect ITag-glycosylation products from complex enzymatic mixtures, even at low concentrations (μM), GlcNAc-ITag **1**, which is a common motif in both type 1 and type 2 HMO chains,7*a* was used as a decoy to hijack glycosyltransferase activity in HBM. β-Linked GlcNAc units are further elongated in HMOs with a β-1,3- or β-1,4-linked galactose unit. Thus **1** was incubated at 37 °C with unpasteurized HBM (1 mg mL^–1^) in the presence of excess UDP-Gal (2.4 equiv.), which was added portion-wise over 5 days, in order to bias the production of galactosylated species (Scheme 2). It is important to highlight that no reaction was observed without addition of the sugar nucleotide (Fig. S3 in ESI†). To our delight, LC-MS analysis of the milk sample showed >86% conversion of **1** to a galactose-containing ITag-disaccharide (M^+^ 675). Importantly, ITag-oligosaccharide products display significantly different retention times in reverse-phase HPLC from other components in HBM (including unlabeled HMOs) to allow their efficient isolation. NMR analysis of the purified ITag-disaccharide confirmed the structure as β-1,4-linked LacNAc-ITag **3**.^18^ No β-1,3-linked galactose containing disaccharide was detected in the mixture, even though it is a common fragment found among HMOs.^19^ This could be attributed to a number of factors *e.g.* low concentrations of the specific enzyme in the milk sample, low activity of the enzyme towards our ITag-substrate or an increased hydrolase activity towards the β-1,3-linked lacto-*N*-biose-ITag dimer.^20^

**Scheme 2:**
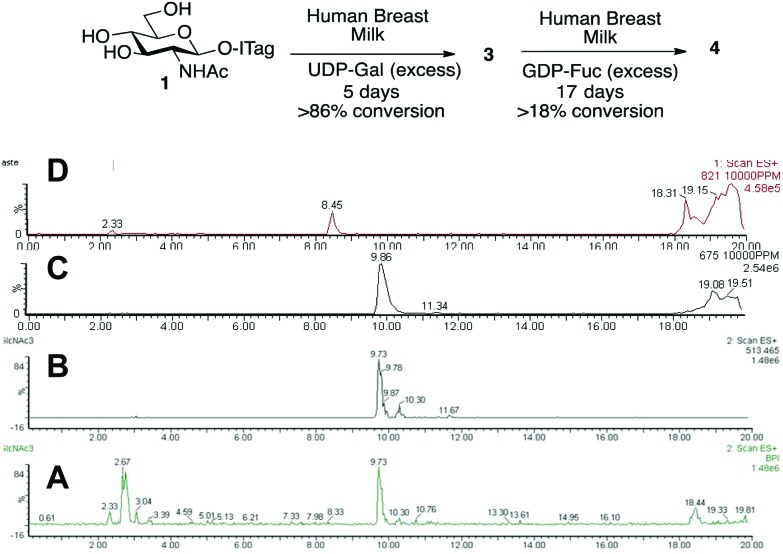
Incubation of GlcNAc-ITag **1** and LacNAc-ITag **3** with UDP-Gal and GDP-Fuc, respectively, in HBM and LC-MS traces of (A) total ion count of HBM incubation of **1** without addition of UDP-Gal; (B) extracted LC-MS trace for **1** (M^+^ 513) before addition of UDP-Gal; (C) extracted LC-MS trace for **3** (M^+^ 675) after 5 days of incubation with **1** and UDP-Gal; (D) extracted LC-MS trace for **4** (M^+^ 821) after 17 days of incubation with **3** and GDP-Fuc.

HMO fucosylation is a common modification, which is mostly dependent on the Lewis blood group of each individual donor. Milk donors can be divided into four different groups depending on their Lewis and secretor gene activity.^21^ The best known examples of fucosyltransferases found in HBM are α-1,2-fucosyltransferase (FUTII)^22^ and α-1,3/4-fucosyltransferase (FUTIII),^23^ which are responsible for the formation of α-1,2- and α-1,3/4-linked fucosides, respectively, and Lewis gene-independent α-1,3-fucosyltransferases.^24^ In order to target HBM fucosyltransferase activity, previously isolated ITag-LacNAc **3**, which has been identified as a substrate for this class of enzymes, was incubated with excess GDP-Fuc in HBM as before (see ESI† for full details). Excitingly, a new monofucosylated ITag-trisaccharide (M^+^ 821) structure was observed by LC-MS analysis, which, after 18 days of incubation and 18% conversion, was isolated. Following NMR characterization, this structure could be assigned to α-1,3-linked ITag-Lewis^x^ derivative **4** (Scheme 2). No other fucosylated species were isolated, which is in agreement with the fucosylation pattern found in HBM for LacNAc dimers9b,^25^ and the known specificity for HBM purified FUTIII.^26^ In addition to fucosyltransferase activity, α-fucosidase activity has also been identified in HBM^27^ and that might be accountable for the lower conversions observed.

Type 1, β-1,3-linked disaccharide lacto-*N*-biose, is also found fucosylated in HBM.^28^ Possible sites for fucosylation include the common α-1,2-linkage to the terminal Gal residue of lacto-*N*-biose, as well as at C4 to form an α-1,4-linkage to the sub-terminal GlcNAc unit; the latter is catalyzed by the same enzyme that transfers a fucose unit to C3 of LacNAc to form Lewis^x^.22*b* Since we did not observe any β-1,3-linked disaccharides in our initial milk incubations with **1**, we decided to synthetically prepare ITag-lacto-*N*-biose **2** (Scheme 3) and explore whether fucosyltransferases activity in the milk could be exploited in this instance. To that end, 4-(halomethyl)-benzenesulfonamide-conjugate **8** 14 was deacetylated using sodium methoxide in methanol. 4,6-*O*-Benzylidene acetal protection, using benzaldehyde dimethylacetal and copper triflate as the catalyst,^29^ produced glycoside acceptor **11** in 77% yield over the two steps, with a 1 : 2 ratio of **11a** : **11b**, ready to be glycosylated at C3. Chemical glycosylation of **11b** with galactose trichloroacetimidate **12** afforded protected lacto-*N*-bioside **13** with complete β-stereocontrol in a 54% yield. Disaccharide **13** was subsequently ITagged by S_N_2 halide displacement with 1-methylimidazole to give **14** in 79% yield. Finally, global deprotection was carried out in two sequential steps: firstly acetal hydrolysis was performed with acetic acid/water, which was followed by acetate removal in the presence of sodium methoxide in methanol to reveal fully unprotected ITag-lacto-*N*-biose **2** in 94% yield.

**Scheme 3:**
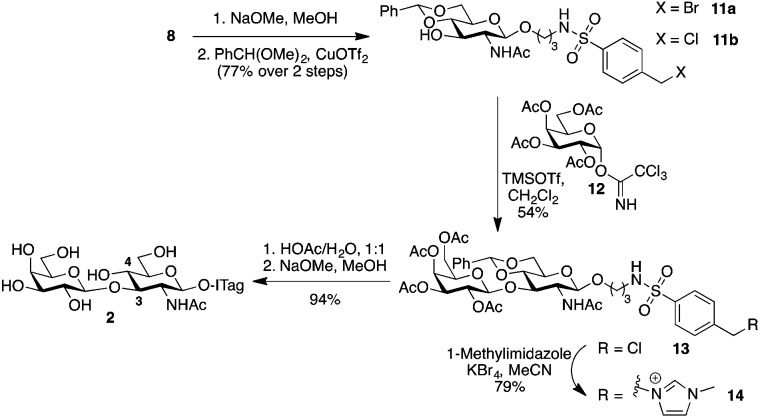
Chemical synthesis of ITag-lacto-*N*-biose **2**.

Milk incubation of **2** in the presence of an excess of GDP-Fuc (3 equiv.) in HBM was carried out as before and the reaction was monitored by LC-MS (Scheme 4). Delightfully, a new trisaccharide species was detected in the LC-MS trace, with a molecular weight consistent with a fucosylated lacto-*N*-biose moiety (M^+^ 821). A maximum conversion of 11% was observed after 16 days of incubation. After HPLC isolation and NMR structural characterization, we were able to assign the new trisaccharide to ITag-Lewis^a^**5** (Fuc-α-1,4-[Gal-β-1,3]-GlcNAc). It is likely that the enzyme responsible for fucosylation of both disaccharides **2** and **3** is α-1,3/4-fucosyltransferase (FUTIII), which has been previously isolated from HBM.^28^,^30^

**Scheme 4:**
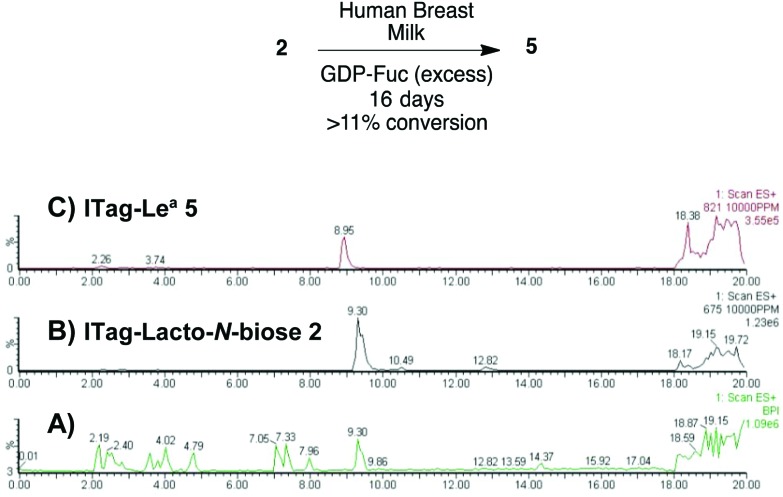
Incubation of ITagged **2** with GDP-Fuc in HBM to obtain ITag-Lewis^a^**5** (M^+^ 821) and LC-MS traces of: (A) total ion count of HBM reaction mixture; (B) extracted LC-MS trace for **2** (M^+^ 675) and (C) extracted LC-MS trace for **5** (M^+^ 821).

## General procedure for HBM incubations

Acceptor (**1**, **2** or **3**) and donor nucleotide (UDP-Gal or GDP-Fuc) were dissolved in HBM, which had been allowed to warm to 37 °C, and the mixture was incubated at 37 °C until no further conversion was observed. Donor nucleotide and fresh milk were added throughout the incubation period and in the case of fucosylated targets, the sample was freeze dried when no further conversion was observed and the reaction re-started by addition of fresh milk and GDP-Fucose to the dried sample (see specific details in ESI†). The reaction was then quenched by cooling on ice and the mixture filtered with ISO-Disc Filters (N 4-2, Nylon 4 mm × 0.2 μm) prior to analysis.

In conclusion, although metabolic oligosaccharide pathways present a number of significant challenges, we have shown that imidazolium-labeled (ITag-) glycosides can be used to harness glycosyltransferase activity directly from human breast milk, without having to isolate the specific enzymes. We demonstrate that ITags covalently attached to glycoside substrates provide a bifunctional chemical handle that can be used to monitor reaction progress by MS, as well as aid in the purification of the products from complex mixtures. Using this technology, we were able to access a series of biologically important oligosaccharides, LacNAc-ITag, ITag-Lewis^x^ and ITag-Lewis^a^, in a matter of days from a readily available source, human breast milk. We believe that these cationic probes offer a practical and novel technology to harness enzyme activity in complex biosynthetic pathways. We believe that the strategy showcased here will find applications in and beyond the field of carbohydrates.

This research was supported by EPSRC CAF EP/L001926/1 and ERC-COG: 648239. We thank Anthony P. Corfield, Matthew J. Watt and Chloe C. Medina for fruitful discussions and Mrs Marion Copeland from The Precious Milk Bank (Southmead Hospital Bristol) for donation of milk samples.

## Supplementary Material

Supplementary information
