# Supplementary material for: Imidazolium-labeled glycosides as probes to harness glycosyltransferase activity in human breast milk
Source: Org Biomol Chem. 2017 Apr 6;15(17):3575–9. doi: 10.1039/c7ob00550d (PMC5708356; doi:10.1039/c7ob00550d)
Supplement: Supplementary file 1 [file OB-015-C7OB00550D-s001.pdf]

Electronic Supplementary Material (ESI) for Organic & Biomolecular Chemistry.
This journal is © The Royal Society of Chemistry 2017

# Electronic Supporting Information

# Imidazolium-labeled Glycosides as Probes to Harness Glycosyltransferase Activity in Human Breast Milk

Imke Sittel[a] and M. Carmen Galan*[a]

## Table of Contents:


| | |
|---|---|
| **General Experimental Procedures** | S2 |
| **Chemical Synthesis of ITag-glycoside acceptors** | S3-S5 |
| **NMR Spectra for novel compounds** | S6-S13 |
| **LC-MS traces for HBM catalyzed reactions and reaction monitoring graphs** | S14-S20 |
| **References** | S21 |

## Experimental

Chemicals and sugar nucleotide donors were purchased from Acros, Aldrich, Carbosynth, Fisher, Jennewein Biotechnologie GmbH, Lancaster and Sigma and used without further purification. Unpasteurised milk samples were donated by The Precious Milk Bank (Southmead Hospital, NHS North Bristol Trust). Milk samples were collected from the human host and immediately frozen and stored frozen at -20 ºC until needed. Anhydrous solvents, where necessary, were obtained through equipment from Anhydrous Engineering (UoB) based on the Grubbs' design. Thin layer chromatography (TLC) was performed on silica gel purchased from Merck (60 $F_{245}$); aromatic species were detected under UV light (254 nm), saccharides were detected using a sulfuric acid solution (10 % in EtOH), followed by charring. Flash chromatography was performed on silica gel purchased from Merck (230-400 mesh (40-63 µm)). NMR spectra were recorded at 25 °C, unless stated otherwise, on either a Varian 400-MHz spectrometer at 400 MHz for the $^{1}$H-nucleus and 101 MHz for the $^{13}$C-nucleus; a Varian 500-MHz spectrometer at 500 MHz for the $^{1}$H-nucleus and 126 MHz for the $^{13}$C-nucleus; a Varian 600-MHz spectrometer at 600 MHz for the $^{1}$H-nucleus and 151 MHz for the $^{13}$C-nucleus or a Varian 700-MHz spectrometer with a cryo probe at 700 MHz for the $^{1}$H-nucleus and 176 MHz for the $^{13}$C-nucleus. As solvents $CDCl_3$, $CD_3OD$ or $D_2O$ have been used. Chemical shifts ($\delta$) have been quoted in parts per million (ppm) to the nearest 0.01 ppm for $^{1}$H- and to the nearest 0.1 ppm for $^{13}$C-spectra. In some cases one decimal was further added to distinguish between two different peaks. The chemical shifts were referenced to the following solvent peaks: $CDCl_3$ ($^{1}$H: $\delta$ = 7.26 ppm (s); $^{13}$C: $\delta$ = 77.2 ppm (t)), $CD_3OD$ ($^{1}$H: $\delta$ = 3.31 ppm (s); $^{13}$C: $\delta$ = 49.0 ppm (sept)), $D_2O$ ($^{1}$H: $\delta$ = 4.79 ppm (s); $^{13}$C: -). Coupling constants ($J$) have been quoted to the nearest 0.1 Hz and, where appropriate, it was further specified as index of coupling constant which protons are involved in the coupling. Other abbreviations used are: b (broad), s (singlet), d (doublet), t (triplet), q (quartet) m (multiplet) or combinations thereof. Assignments of the recorded signals were made, where necessary, with the aid of COSY, TOCSY, HSQC and HMBC experiments. MALDI-TOF spectra were recorded using an Applied Biosystems 4700 Proteomics analyzer MALDI-TOF spectrometer; 2,5-dihydroxybenzoic acid in MeCN was used as matrix. High-resolution ESI spectra were recorded by the University of Bristol mass spectrometry service. Optical rotations were measured on a Bellingham + Stanley ADP220 polarimeter. The units of the specific rotation, (deg·ml)/(g·dm) are implicit and are not included with the reported value; the concentration *c* is given in g/100 mL. LC-MS spectra were recorded on either a Waters 2795 HT Alliance Separation Module High Performance Liquid Chromatography (*Module A*) machine with a Waters Micromass ZQ mass spectrometer and a Waters photodiode array detector; a Waters 2545 Binary Gradient Module (*Module B*) with a Waters System Fluid Organizer, a Waters photodiode array detector and a Waters Micromass Quattro micro API or an LC Packings Famos (*Module C*) system with a Bruker esquire 6000 mass spectrometer. Samples for reaction monitoring were run on Module A with a 05-15 % MeOH in water gradient (containing 0.05 % formic acid; *gradient A*), on Module B with a 15-50 % MeOH in water gradient (containing 0.05 % formic acid; *gradient B*) or on Module C with a 5-95 % MeOH in water gradient (containing 0.1 % formic acid; *gradient C*). Preparative HPLC was performed on a Grace Reveleris Prep Purification System with UV and ELS detector, using a 5-8 % MeOH in water gradient (containing 0.05 % formic acid; *gradient D*) for disaccharide **3** or using 4 % isocratic MeOH in water (containing 0.05 % formic acid; *gradient E*) for trisaccharides **4** and **9**.

**3-(4-(Halomethyl)benzenesulfonamidyl) propyl 2-acetamido-4,6-benzylidene acetal-2-deoxy-*β*-D-glucopyranoside** (**11**) Sulfonamide **8** (94 mg, 0.16 mmol) was dissolved in a solution of NaOMe in MeOH (0.1 M, 1 ml) and stirred for 2 h at room temperature. Upon consumption of starting material, as observed by TLC ($CH_2Cl_2$/MeOH, 9:1), the reaction mixture was neutralized over Celite $H^+$ resin and concentrated, to afford the unprotected intermediate, which was taken up immediately in anhydrous MeCN (1 ml). Copper triflate (16 mg, 0.044 mmol) and benzaldehyde dimethyl acetal (44 µl, 0.30 mmol) were added. The reaction mixture was then sonicated at room temperature under $N_2$ for 30 min, until TLC ($CH_2Cl_2$/MeOH, 9:1) showed reaction completion. The mixture was concentrated and purified *via* flash column chromatography ($CH_2Cl_2$/MeOH, 1:0 → 19:1), to

afford **11** with a bromide/chloride ratio of 1:2 as a colorless solid (68 mg, 77 % over 2 steps). $^1$H-NMR (400 MHz, $CD_3OD$): $\delta$ = 7.87 (2H, d, $J$ = 8.5 Hz, Ar), 7.64 (2H, d, $J$ = 8.1 Hz, Ar), 7.54-7.48 (2H, m, Ar), 7.38-7.33 (3H, m, Ar), 5.61 (1H, s, C*H*Ph), 4.74 (2H, s, C$H_2$Cl), 4.65 (2H, s, C$H_2$Br), 4.52-4.48 (1H, m, H-1), 4.28 (1H, dd, $J_{6a,6b}$ = 10.3, $J_{6a,5}$ = 4.9 Hz, H-6a), 3.88-3.81 (1H, m, OC*H*H), 3.80-3.73 (3H, m, H-2, H-4, H-6b), 3.58-3.49 (2H, m, H-3, OCH*H*), 3.47-3.39 (1H, m, H-5), 3.01-2.89 (2H, m, C$H_2$NH), 1.97 (3H, s, C$H_3$, NHAc), 1.75-1.66 (2H, m, $OCH_2CH_2$) ppm; $^{13}$C-NMR (126 MHz, $CD_3OD$): $\delta$ = 173.8 (*C*=O), 144.0, 141.8, 139.1 (3 × $C_q$, Ar), 130.4, 129.9, 129.0, 128.4, 127.5 (5 × *C*H, Ar), 103.3 (C-1), 103.0 (*C*HPh), 82.9 (C-3), 72.6 (C-4), 69.7 (C-6), 68.0 (O*C*$H_2$), 67.7 (C-5), 58.0 (C-2), 45.7 (*C*$H_2$Cl), 41.3 (*C*$H_2$NH), 32.3 (*C*$H_2$Br), 30.9 ($OCH_2CH_2$), 23.1 (*C*$H_3$, NHAc) ppm; ESI-HRMS for $C_{25}H_{31}N_2O_8S^{35}Cl^+(M^+)$: calculated: 577.1387; found: 577.1388; $C_{25}H_{31}N_2O_8S^{79}BrNa^+(MNa^+)$: calculated: 621.0877; found: 621.0896.

**3-(4-(Chloromethyl)benzenesulfonamidyl) propyl 2',3',4',6'-tetra-*O*-acetyl-*β*-D-galactopyranosyl-(1–3)-2-acetamido-4,6-benzylidene acetal-2-deoxy-*β*-D-glucopyranoside (13)** Pre-dried sulfonamide linked glucoside **11b** (38 mg, 0.069 mmol) and trichloroacetimidate **12** (63 mg, 0.13 mmol) were dissolved in anhydrous $CH_2Cl_2$ (30 ml), before activated 4 Å molecular sieves were added. The reaction mixture was left to stir at room temperature under $N_2$ for 30 min, before it was cooled to 0 °C and TMSOTf (3.0 µl, 0.02 mmol) was added. The mixture was then allowed to warm to room temperature and left to stir for 6 h, until TLC (EtOAc/*n*-hexane, 5:1) showed consumption of starting material. The mixture was then quenched with $Et_3N$ (10 µl) at 0 °C, filtered through Celite, concentrated and purified *via* flash column chromatography (EtOAc/*n*-hexane, 2:3 → 1:0), to afford **13** as a colorless solid (33 mg, 54 %). $^1$H-NMR (400 MHz, $CDCl_3$): $\delta$ = 7.85-7.80 (2H, m, Ar), 7.57-7.52 (2H, m, Ar), 7.49-7.44 (2H, m, Ar), 7.40-7.34 (3H, m, Ar), 6.14 (1H, d, $J$ = 7.2 Hz, N*H*Ac), 5.52 (1H, s, C*H*Ph), 5.32-5.28 (2H, m, H-4', N*H*$SO_2$), 5.18 (1H, dd, $J_{2',3'}$ = 10.4, $J_{2',1'}$ = 8.0 Hz, H-2'), 5.09 (1H, d, $J_{1,2}$ = 8.2 Hz, H-1), 4.93 (1H, dd, $J_{3',2'}$ = 10.4, $J_{3',4'}$ = 3.4 Hz, H-3'), 4.71 (1H, d, $J_{1',2'}$ = 8.0 Hz, H-1'), 4.55 (1H, dd, $J_{3,2}$ = 10.0, $J_{3,4}$ = 8.9 Hz, H-3), 4.49 (2H, s, C$H_2$Cl), 4.32 (1H, dd, $J_{6a,6b}$ = 10.4, $J_{6a,5}$ = 4.8 Hz, H-6a), 4.10-4.05 (1H, m, OC*H*H), 3.97-3.90 (2H, m, H-6a', OCH*H*), 3.76 (1H, t, $J_{6b,5}$ = $J_{6b,6a}$ = 10.3 Hz, H-6b), 3.69-3.61 (3H, m, H-4, H-5', H-6b'), 3.52 (1H, td, $J_{5,4}$ = $J_{5,6b}$ = 9.7, $J_{5,6a}$ = 4.9 Hz, H-5), 3.25-3.17 (1H, m, H-2), 3.12-3.06 (2H, m, C$H_2$NH), 2.11, 2.03, 2.02, 1.96, 1.95 (15H, all s, 5 × C$H_3$, Ac), 1.77-1.71 (2H, m, $OCH_2CH_2$) ppm; $^{13}$C-NMR (126 MHz, $CDCl_3$): $\delta$ = 171.5, 170.4, 170.34, 170.26, 169.7, (5 × *C*=O), 142.7, 140.1, 137.1 (3 × $C_q$, Ar), 129.9, 129.4, 128.5, 127.6, 126.2 (5 × *C*H, Ar), 101.5 (*C*HPh), 100.7 (C-1'), 100.0 (C-1), 80.4 (C-4), 77.2 (C-3), 71.2 (C-3'), 70.6 (C-5'), 69.5 (C-2'), 68.8 (C-6), 68.2 (O*C*$H_2$), 67.0 (C-4'), 66.2 (C-5), 61.1 (C-6'), 58.3 (C-2), 41.2 (*C*$H_2$NH), 31.3 (*C*$H_2$Cl), 29.0 ($OCH_2CH_2$), 23.8 (*C*$H_3$, NHAc), 20.9, 20.80, 20.76, 20.7 (4 × *C*$H_3$, Ac) ppm; ESI-HRMS for $C_{39}H_{49}{}^{35}ClN_2O_{17}SNa^+(MNa^+)$: calculated: 907.2338; found: 907.2377; $[\alpha]^{23}_{D}$ = -17.8 (*c* 0.42, MeOH).

**3-(4-(3-Methylimidazolium)methylbenzenesulfonamidyl) propyl 2',3',4',6'-tetra-*O*-acetyl-*β*-D-galactopyranosyl-(1–3)-2-acetamido-4,6-benzylidene acetal-2-deoxy-*β*-D-glucopyranoside (14)** Sulfonamide **13** (25 mg, 0.028 mmol) and $KBF_4$ (18 mg, 0.14 mmol) were dissolved in anhydrous MeCN (1 ml), before 1-methylimidazole (9.3 µl, 0.12 mmol) was added. The reaction mixture was heated to 80 °C under $N_2$ and stirred for 18 h, until TLC (acetone/toluene, 2:1) showed consumption of the starting material. The reaction mixture was concentrated and washed with EtOAc (3 × 2 ml), to afford **14** as a colorless solid (22 mg, 79 %). $^1$H-NMR (400 MHz, MeOD): $\delta$ = 9.04 (1H, s, NC*H*N), 7.91 (2H, d, $J$ = 8.3 Hz, Ar), 7.67 (1H, d, $J$ = 2.0 Hz, Ar), 7.64-7.59 (3H, m, Ar), 7.54-7.50 (2H, m, Ar), 7.40-7.31 (3H, m, Ar), 5.58 (1H, s, C*H*Ph), 5.54 (2H, s, C$H_2$Ar), 5.31 (1H, bd, $J_{4,3/5}$ = 2.2 Hz, H-4'), 5.08-5.05 (2H, m, H-2', H-3'), 4.83-4.79 (1H, m, H-1), 4.46 (1H, d, $J_{1',2'}$ = 8.3 Hz, H-1'), 4.24 (1H, dd, $J_{6a,6b}$ = 10.4, $J_{6a,5}$ = 4.9 Hz, H-6a), 4.18-4.07 (1H, m, H-6a'), 3.99-3.74 (9H, m (s at 3.94 (3H, N$CH_3$)), H-2, H-3, H-5', H-6b, H-6b', OC*H*H), 3.61 (1H, t, $J_{4,3}$ = $J_{4,5}$ = 9.2 Hz, H-4), 3.55-3.48 (1H, m, OCH*H*), 3.43 (1H, td, $J_{5,4}$ = $J_{5,6b}$ = 9.8, $J_{5,6a}$ = 5.0 Hz, H-5), 2.98-2.85 (2H, m, C$H_2$NH), 2.07, 2.06, 1.97, 1.92 (× 2) (15H, all s, 5 × C$H_3$, Ac), 1.72-1.63 (2H, m, $OCH_2CH_2$) ppm; $^{13}$C-NMR (126 MHz, MeOD): $\delta$ = 173.2, 171.91, 171.89, 171.54, 171.46, (5 × *C*=O), 142.6, 139.9, 139.1 (3 × $C_q$, Ar), 130.3, 129.9, 129.1, 129.0, 127.4 (5 × *C*H, Ar), 125.5, 123.9 (2 × *C*H, Im), 103.2 (C-1'), 102.2 (*C*HPh), 102.0 (C-1), 81.0 (C-4), 79.7 (C-3), 72.5 (C-3'), 71.5 (C-5'), 70.6 (C-2'), 69.6 (C-6), 68.6 (C-4'), 67.9 (O*C*$H_2$), 67.5 (C-5), 62.2 (C-6'), 56.2 (C-2), 53.2 (*C*$H_2$N), 41.2

($CH_2$NH), 36.7 ($CH_3$N), 30.7 ($OCH_2CH_2$), 23.3 ($CH_3$, NHAc), 21.0, 20.6, 20.49, 20.48 (4 × $CH_3$, Ac) ppm; ESI-HRMS for $C_{43}H_{55}N_4O_{17}S^+$($M^+$): calculated: 931.3277; found: 931.3286; $|\alpha|_D^{20}$ = -42.9 (*c* 0.16, MeOH).

**3-(4-(3-Methylimidazolium)methylbenzenesulfonamidyl) propyl *β*-D-galactopyranosyl-(1–3)-2-acetamido-2-deoxy-*β*-D-glucopyranoside (2)** ITagged disaccharide **14** (8 mg, 9 µmol) was dissolved in a 1:1 mixture of HOAc/$H_2O$ (1 ml) and stirred at 45 °C for 18 h, until MALDI showed consumption of starting material. The mixture was concentrated and taken up in MeOH. NaOMe solution was added and the mixture was stirred for 2 h at room temperature, until MALDI showed consumption of starting material. The mixture was then neutralized over Celite $H^+$ resin, filtered and concentrated, to afford **2** as a colorless solid (6.31 mg, 94 %). $^1$H-NMR (500 MHz, $D_2O$): $\delta$ = 8.72 (1H, s, NC*H*N), 7.80 (2H, d, $J$ = 8.1 Hz, Ar), 7.48 (2H, d, $J$ = 8.0 Hz, Ar), 7.39-7.37 (2H, m, NC*H*C*H*N), 5.41 (2H, s, $CH_2$Ar), 4.38 (1H, d, $J_{1,2}$ = 7.5 Hz, H-1), 4.30 (1H, d, $J_{1',2'}$ = 7.9 Hz, H-1’), 3.82-3.74 (6H, m (s at 3.78 (3H, N$CH_3$)), H-4’, H-6a, OC*H*H), 3.69-3.57 (6H, m, H-2, H-3, H-4, H-6a’, H-6b, H-6b’), 3.55-3.45 (2H, m, H-3’, OCH*H*), 3.43-3.28 (3H, m, H-2’, H-5, H-5’), 2.90-2.79 (2H, m, $CH_2$NH), 1.81 (3H, s, $CH_3$, NHAc), 1.63-1.53 (2H, m, $OCH_2CH_2$) ppm; $^{13}$C-NMR (126 MHz, $D_2O$): $\delta$ = 174.3 (*C*=O), 139.1, 138.9 (2x Cq, Ar), 136.4 (NCHN), 129.2, 127.5, 124.0, 122.4 (4 × *C*H, Ar), 103.5 (C-1’), 100.7 (C-1), 82.4 (C-3), 75.3 (C-5’), 75.2 (C-4), 72.4 (C-3’), 70.6 (C-2’), 68.7 (C-5), 68.5 (C-4’), 67.1 (O$CH_2$), 61.0 (C-6), 60.7 (C-6’), 54.4 (C-2), 51.9 ($CH_2$Ar), 39.6 ($CH_2$NH), 35.8 (N$CH_3$), 28.7 ($OCH_2CH_2$), 22.1 ($CH_3$, NHAc) ppm; ESI-HRMS for $C_{28}H_{43}N_4O_{13}S^+$($M^+$): calculated: 675.2542; found: 675.2541.

***General procedure for HBM incubations*:** Acceptor (**1**, **2** or **3**) and donor nucleotide (UDP-Gal or GDP-Fuc) were dissolved in HBM, which had been allowed to warm to 37 ºC, and the mixture was incubated at 37 °C until no further conversion was observed. Donor nucleotide and fresh milk were added throughout the incubation period (see specific details in each example). The reaction was then quenched by cooling on ice and the mixture filtered with ISO-Disc Filters (N 4-2, Nylon 4 mm x 0.2 um) prior to analysis.

**3-(4-(3-Methylimidazolium)methylbenzenesulfonamidyl) propyl *β*-D-galactopyranosyl-(1–3)-2-acetamido-2-deoxy-*β*-D-glucopyranoside (3)** *Following the general procedure for HBM incubations* acceptor **1** (2.40 mg, 4.00 µmol) and UDP-Gal (2.44 mg, 4.00 µmol) were incubated with HBM (1.3 ml) for 100 h. After 27, 44 and 92 h 1 equiv. of donor was added, until the reaction was quenched after 100 h (4 d), at a conversion of 85 %, as estimated by LC-MS. The crude mixture was purified *via* preparative RP HPLC (*gradient D*), to afford **3** as colourless solid (1.36 mg, 45 %). *Spectroscopic data was in agreement with that reported in the literature.*[1]

**3-(4-(3-Methylimidazolium)methylbenzenesulfonamidyl) propyl *α*-L-fucopyranosyl-(1–4)-(*β*-D-galactopyranosyl)-(1–3)-2-acetamido-2-deoxy-*β*-D-glucopyranoside (5)** *Following the general procedure for HBM incubations* acceptor **2** (5.90 mg, 7.74 µmol) and GDP-Fuc donor (1.63 mg, 2.58 µmol) were incubated in HBM (5 ml). After 2, 5, 7 and 8 d, 0.25-0.50 equiv. of donor were added. After 9 d the reaction was quenched. The crude mixture was then centrifuged and the supernatant freeze-dried. The incubation was restarted with fresh HBM (5 ml) and 0.35 equiv. of donor. After a further 4 d another 0.25 equiv. of donor was added. The incubation was again quenched after 5 d, following the same procedure as described above and restarted with fresh HBM (5 ml) and 0.20 equiv. of donor. After a further 1 d another 0.20 equiv. of donor was added. The incubation was quenched for a final time at a conversion of 11 %, as estimated by LC-MS. The crude mixture was purified *via* preparative RP-HPLC (*gradient E*), to afford **5** as colorless solid (0.49 mg, 7 %). $^1$H-NMR (700 MHz, $D_2O$, 30 °C): $\delta$ = 8.89 (1H, s, NC*H*N), 7.98 (2H, d, $J$ = 8.1 Hz, Ar), 7.66 (2H, d, $J$ = 8.1 Hz, Ar), 7.56 (2H, bs, Im), 5.59 (2H, s, $CH_2$Ar), 5.07 (1H, d, $J_{1'',2''}$ = 4.0 Hz, H-1''), 4.92 (1H, q, $J$ = 6.7 Hz, H-5''), 4.53 (1H, d, $J_{1',2'}$ = 7.6 Hz, H-1'), 4.53 (1H, d, $J_{1,2}$ = 8.5 Hz, H-1), 4.10 (1H, t, $J_{3,2}$ = $J_{3,4}$ = 9.7 Hz, H-3), 4.02 (1H, dd, $J_{6a,6b}$ = 12.3, $J_{6a,5}$ = 2.4 Hz, H-6a), 4.00-3.84 (10H, m, H-2, H-6b, H-4', H-2'', H-3'', H-4'', OC*H*H, N$CH_3$), 3.81-3.74 (3H, m, H-4, H-6a', H-6b'), 3.68 (1H, dd, $J_{3',2'}$ = 9.8, $J_{3',4'}$ = 3.5 Hz, H-3'), 3.67-3.61 (2H, m, H-5', OCH*H*), 3.59 (1H, ddd, $J_{5,4}$ = 9.5, $J_{5,6b}$ = 4.3, $J_{5,6a}$ = 2.4 Hz, H-5), 3.54 (1H, dd, $J_{2',3'}$ = 9.8, $J_{2',1'}$ = 7.6 Hz, H-2'), 3.03 (2H, tt, $J$ = 12.9, $J$ = 6.5 Hz, $CH_2$NH), 1.99 (3H, s, NH*Ac*), 1.79-1.72 (2H, m, $OCH_2CH_2$), 1.24 (3H, d, $J_{6'',5''}$ = 6.6 Hz, H-6'') ppm; $^{13}$C-NMR (176 MHz, $D_2O$, 30 °C): $\delta$ = 174.2 (C=O), 139.2, 139.0 (2 × $C_q$, Ar), 135.9 (NCHN), 129.2, 127.6

(2 × *C*H, Ar), 123.0 (*C*H, Im), 102.7 (C1'), 100.7 (C1), 97.8 (C1''), 76.0 (C3), 75.3 (C-5), 74.8 (C-5'), 72.31 (C-4), 72.25 (C-3'), 71.9, 70.4 (C-2'), 68.7, 68.4, 67.6, 67.1 (O*C*$H_2$), 66.7 (C-5''), 61.5 (C-6), 59.6 (C-6'), 55.6 (C-2), 51.9 (*C*$H_2$Ar), 39.5 (*C*$H_2$NH), 35.6 (N*C*$H_3$), 28.7 ($OCH_2$*C*$H_2$), 22.1 (*C*$H_3$, Ac), 15.2 (C-6''); ESI-HRMS for $C_{34}H_{53}N_4O_{17}S^+$($M^+$): calculated: 821.3121; found: 821.3126.

**3-(4-(3-Methylimidazolium)methylbenzenesulfonamidyl) propyl *α*-L-fucopyranosyl-(1–3)-(*β*-D-galactopyranosyl)-(1–4)-2-acetamido-2-deoxy-*β*-D-glucopyranoside (4)** *Following the general procedure for HBM incubations* acceptor **3** (9.70 mg, 12.7 μmol) and GDP-Fuc donor (3.26 mg, 5.15 μmol) were incubated in HBM (10 ml). After 1, 2, and 5 d, 0.40-0.60 equiv. of donor were added. After 7 d the reaction was quenched. The crude mixture was centrifuged and the supernatant freeze-dried. The incubation was restarted with HBM (10 ml) and 0.30 equiv. of donor. After a further 2 and 6 d another 0.25 equiv. of donor was added. After 8 d the incubation was again quenched, following the same procedure as described above and restarted with HBM (10 ml) and 0.25 equiv. of donor. After a further 1 d 0.25 equiv. of donor was added. The incubation was quenched for a final time after 2 d at a conversion of 18 %, as estimated by LC-MS. The crude mixture was purified *via* preparative RP HPLC (*gradient E*), to afford **4** as colorless solid (0.72 mg, 6 %). *Spectroscopic data was in agreement with that reported in the literature.*[1]

**NMR Spectra**

Compound **11**

## $^{1}$H-NMR

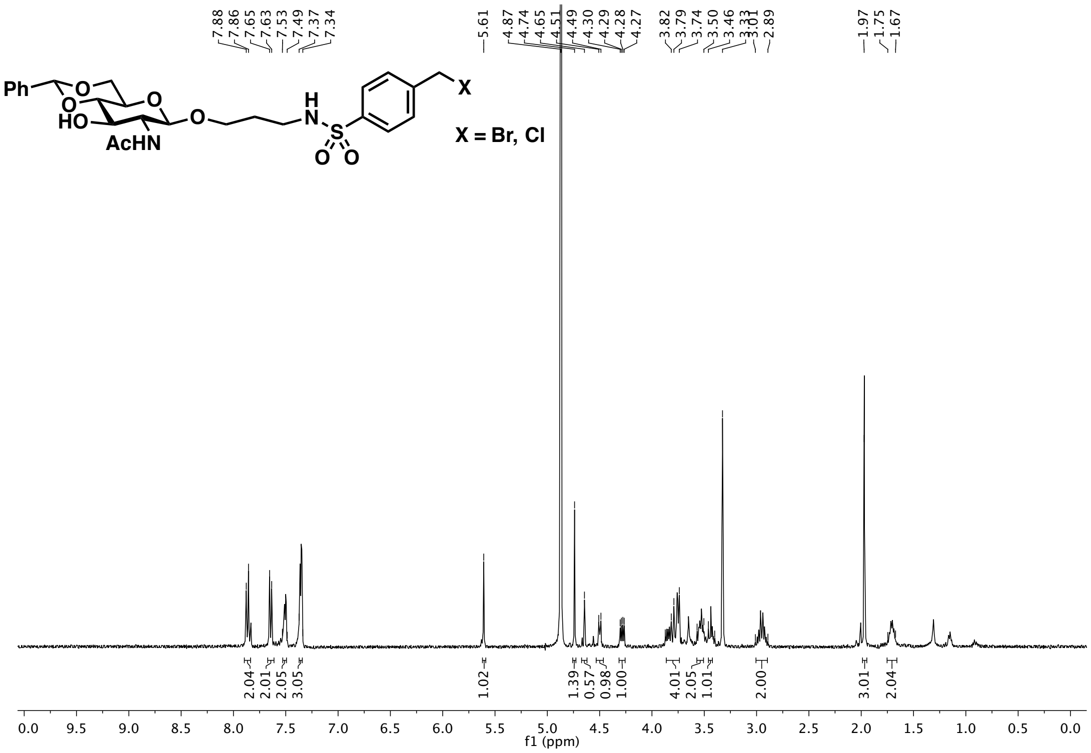


## $^{13}$C-NMR

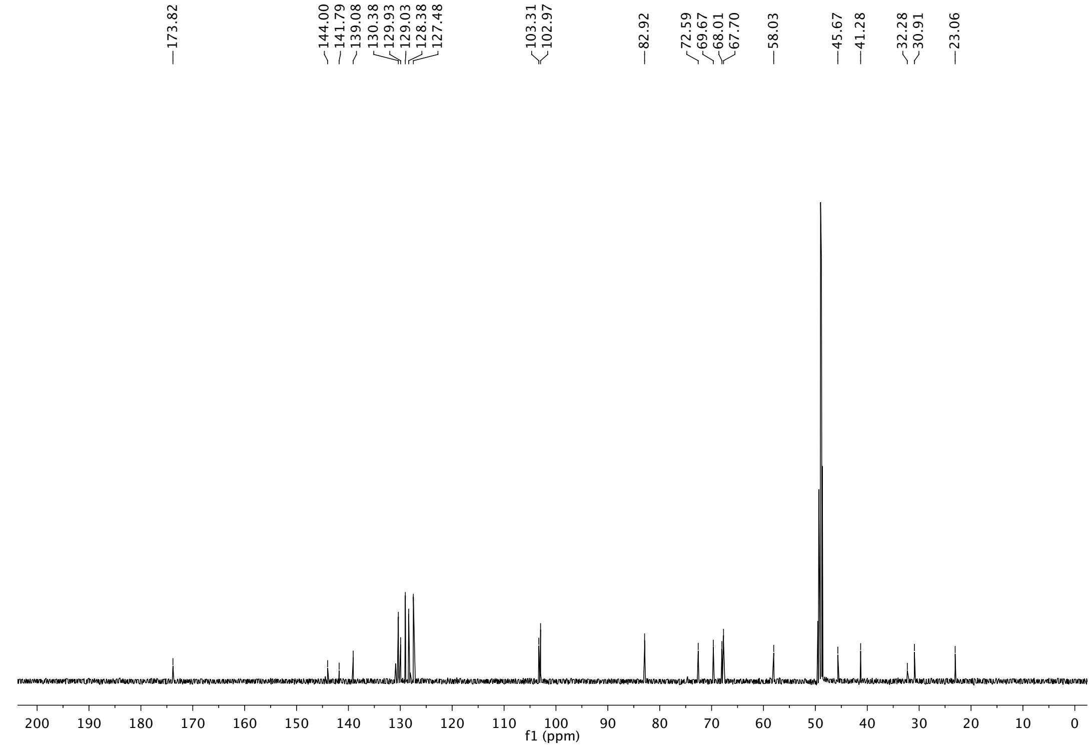

Compound **13**

## $^{1}$H-NMR

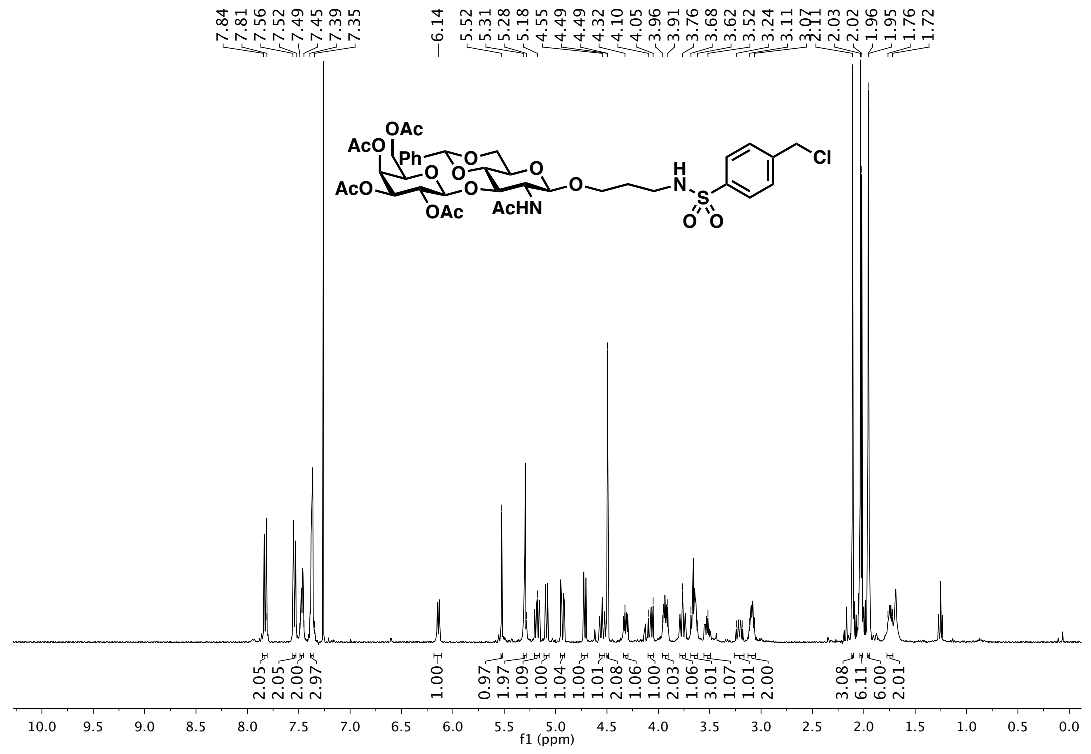

## $^{13}$C-NMR

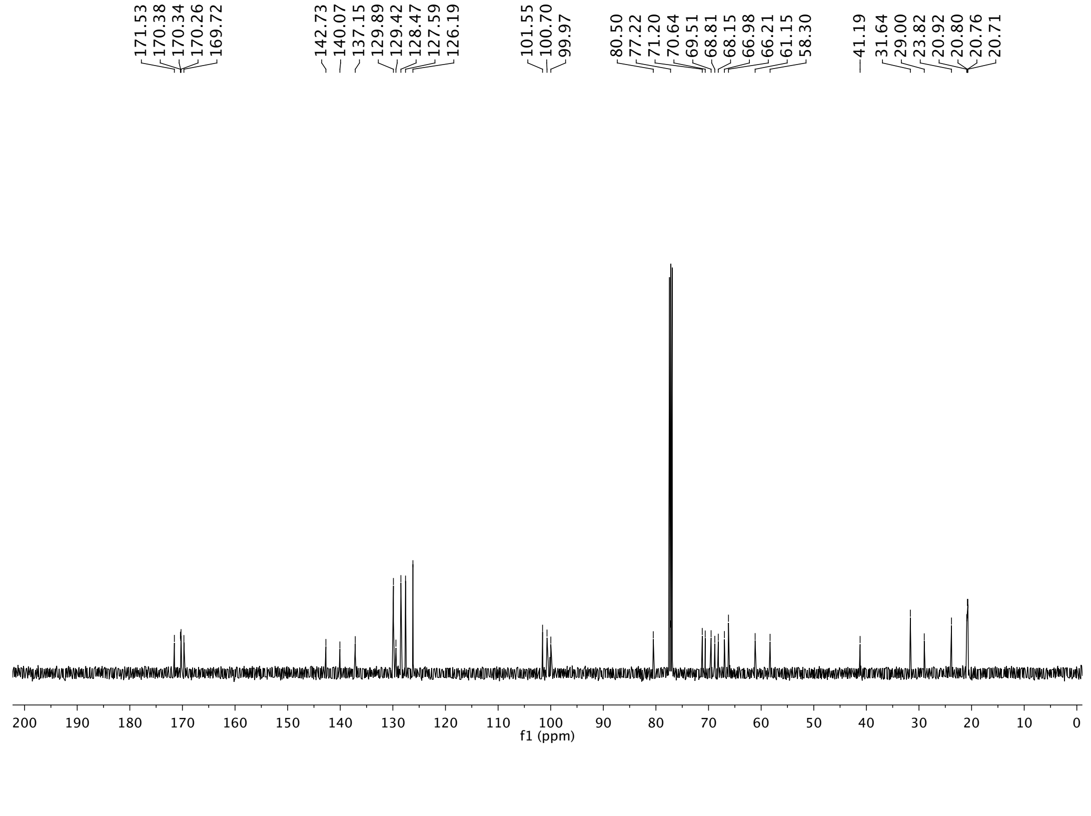

Compound **14**

**$^{1}$H-NMR**

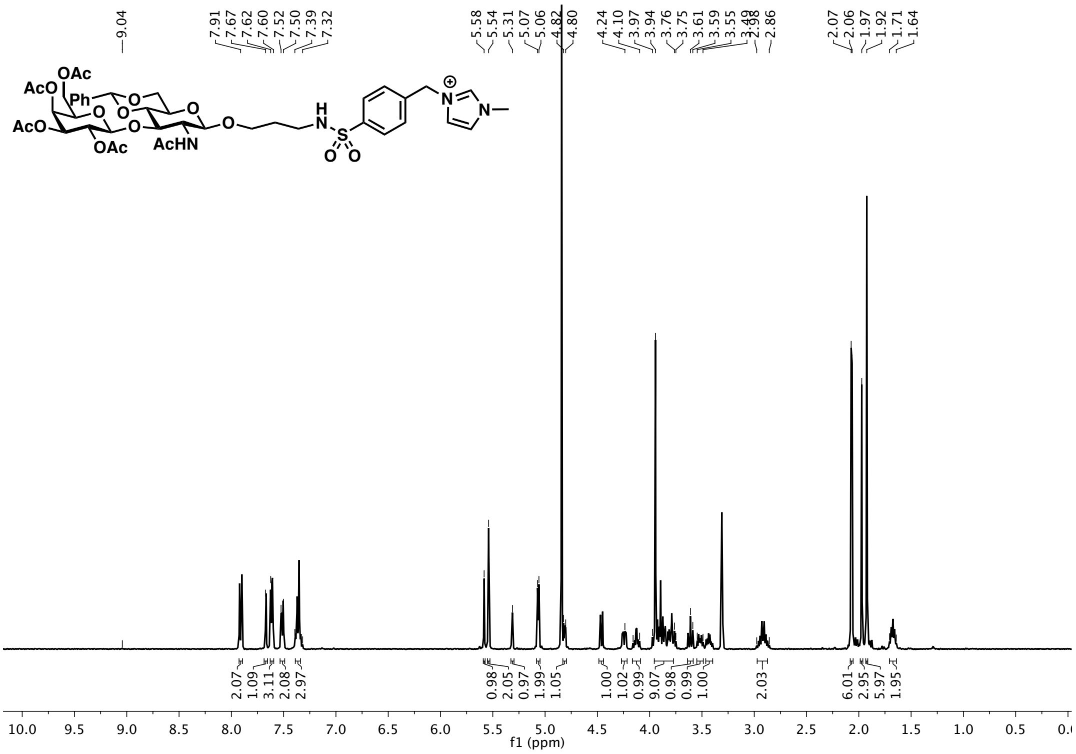


**$^{13}$C-NMR**

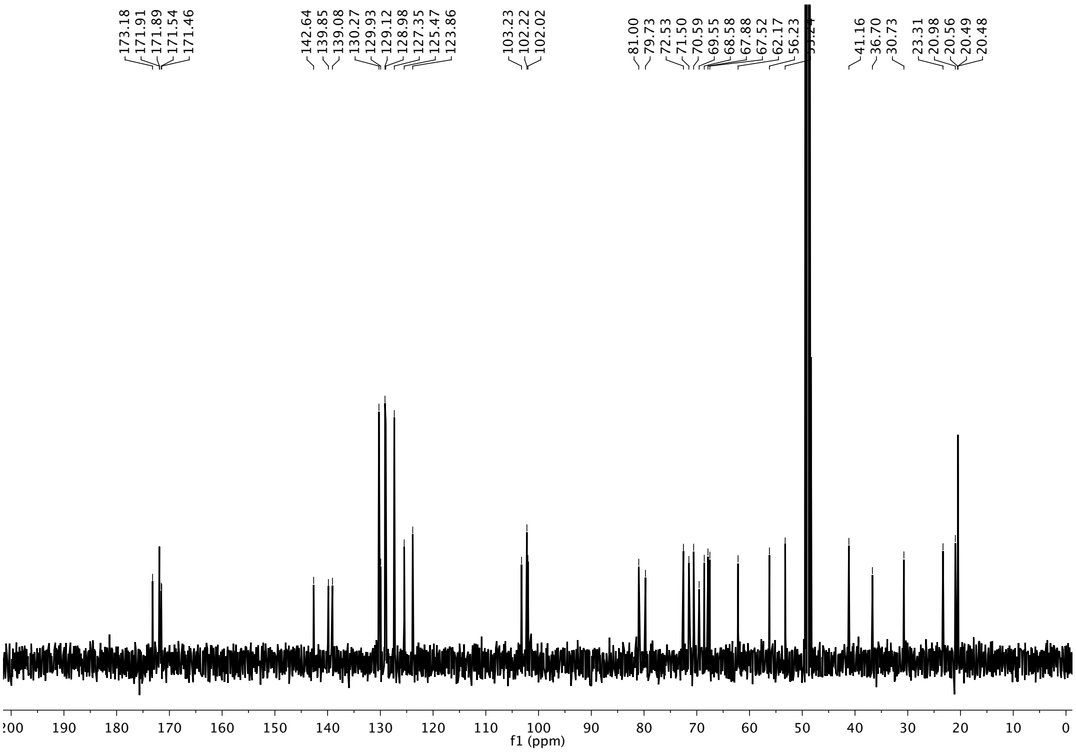

Compound **2**

## $^{1}$H-NMR

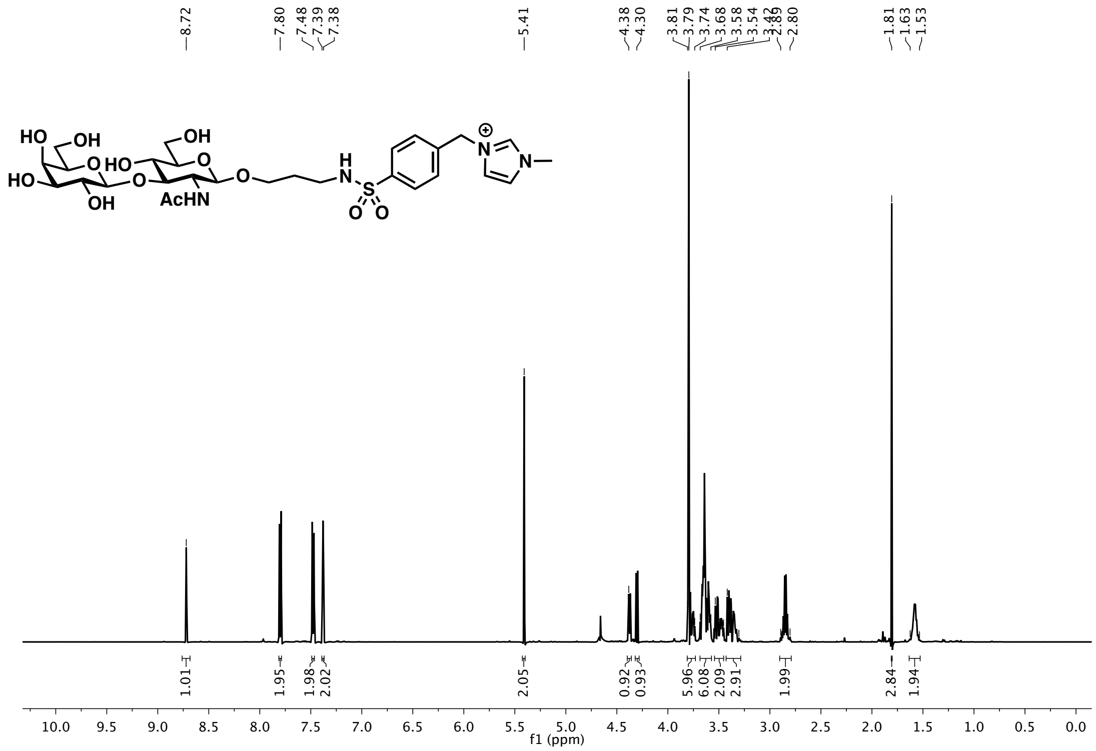


## $^{13}$C-NMR

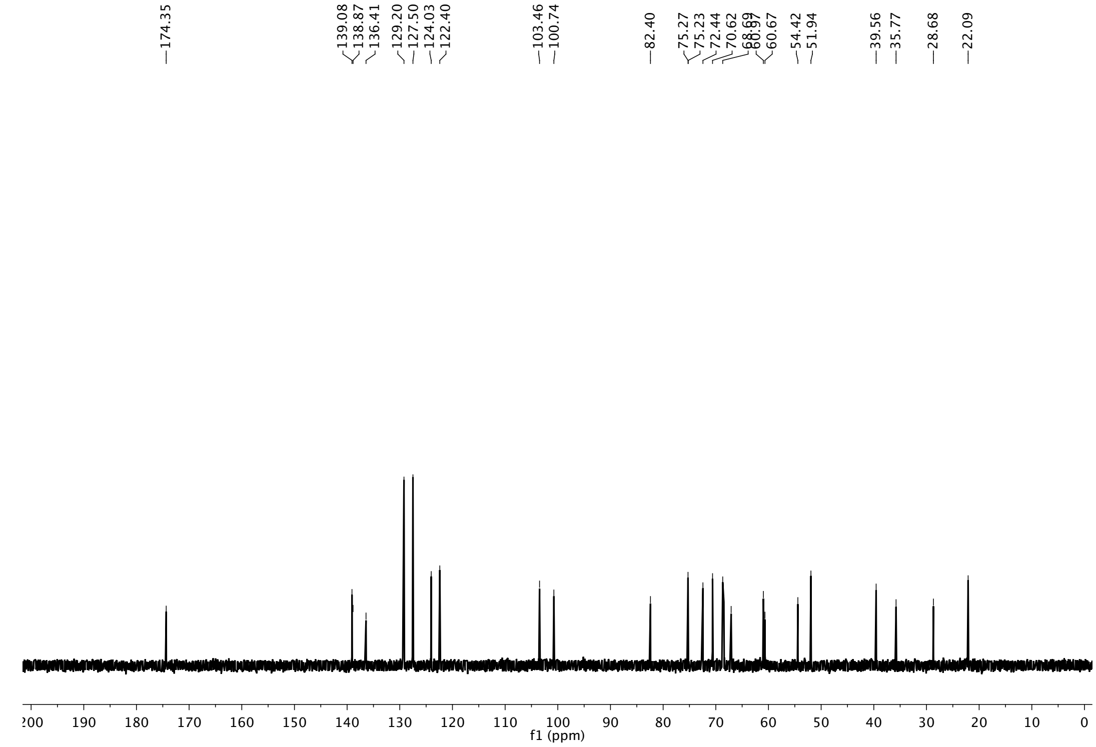

Compound **3**

## $^1$H-NMR

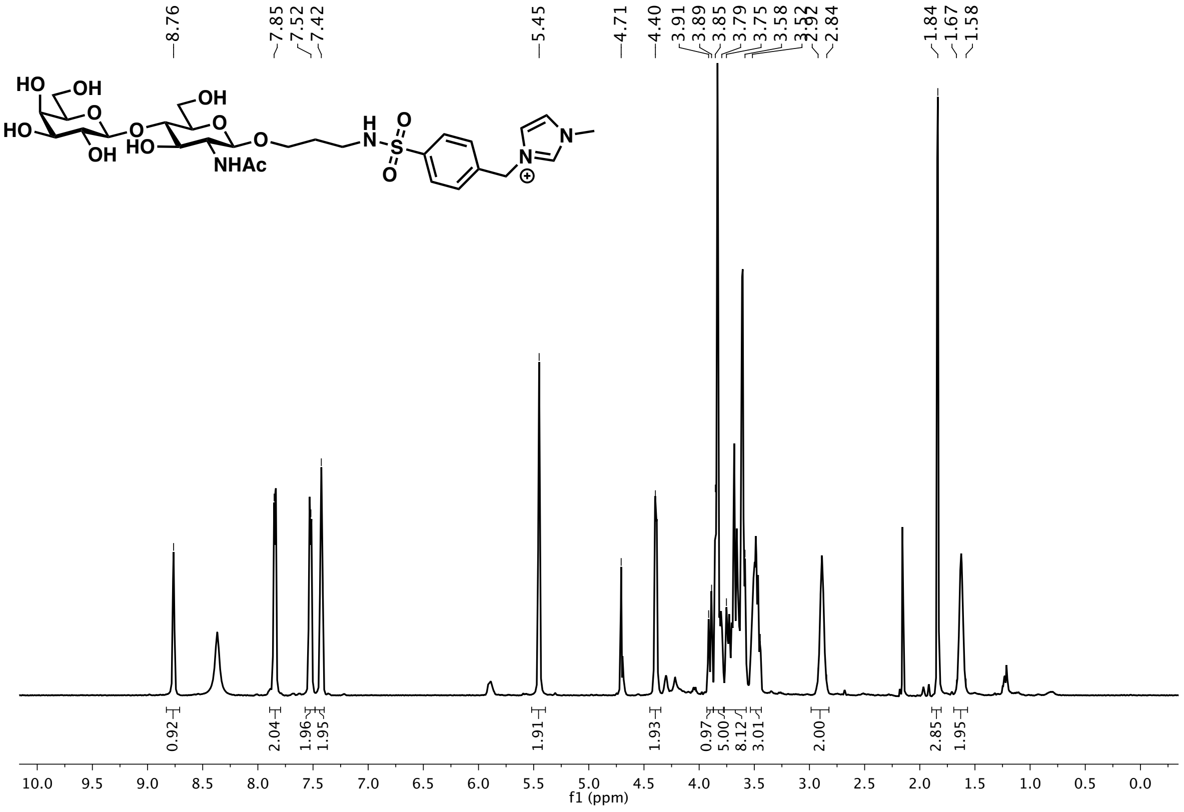


## $^{13}$C-NMR

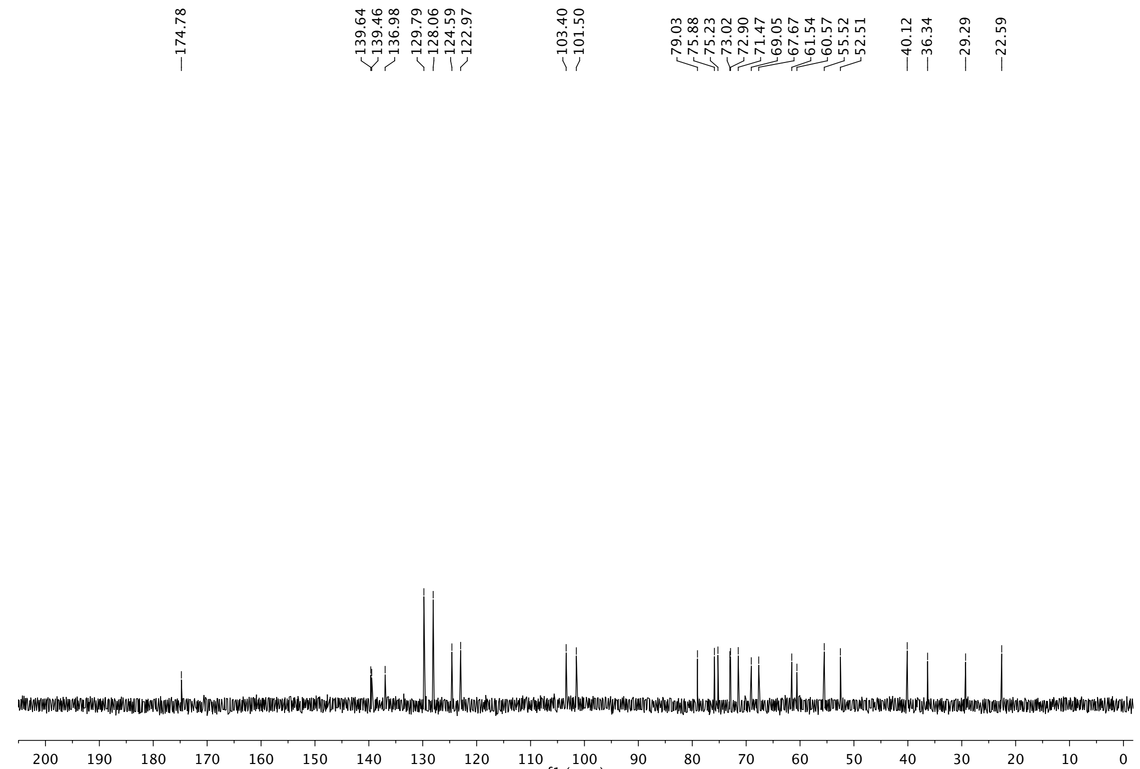

Compound **4**

**$^1$H-NMR**

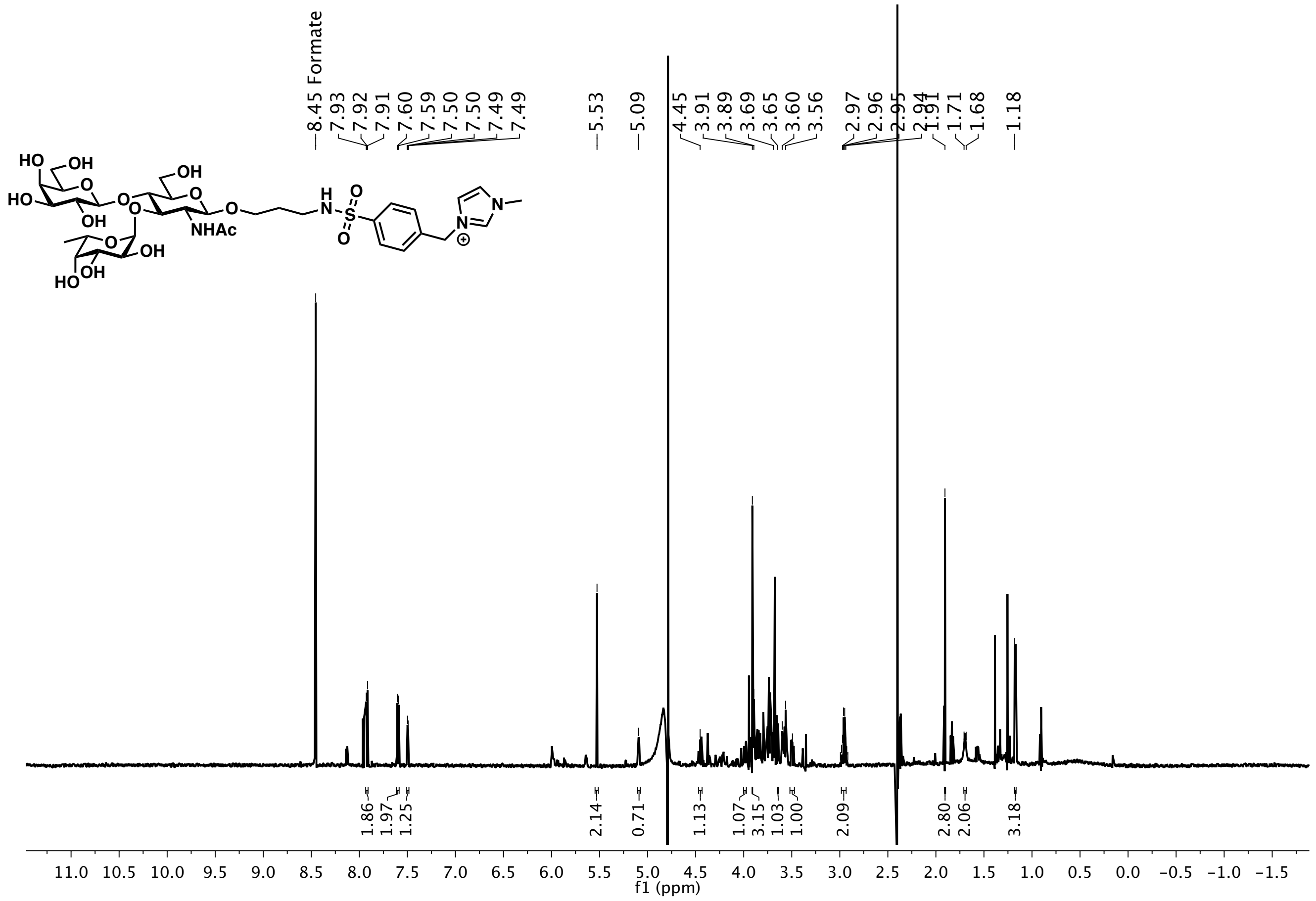

Compound **5**

## $^{1}$H-NMR

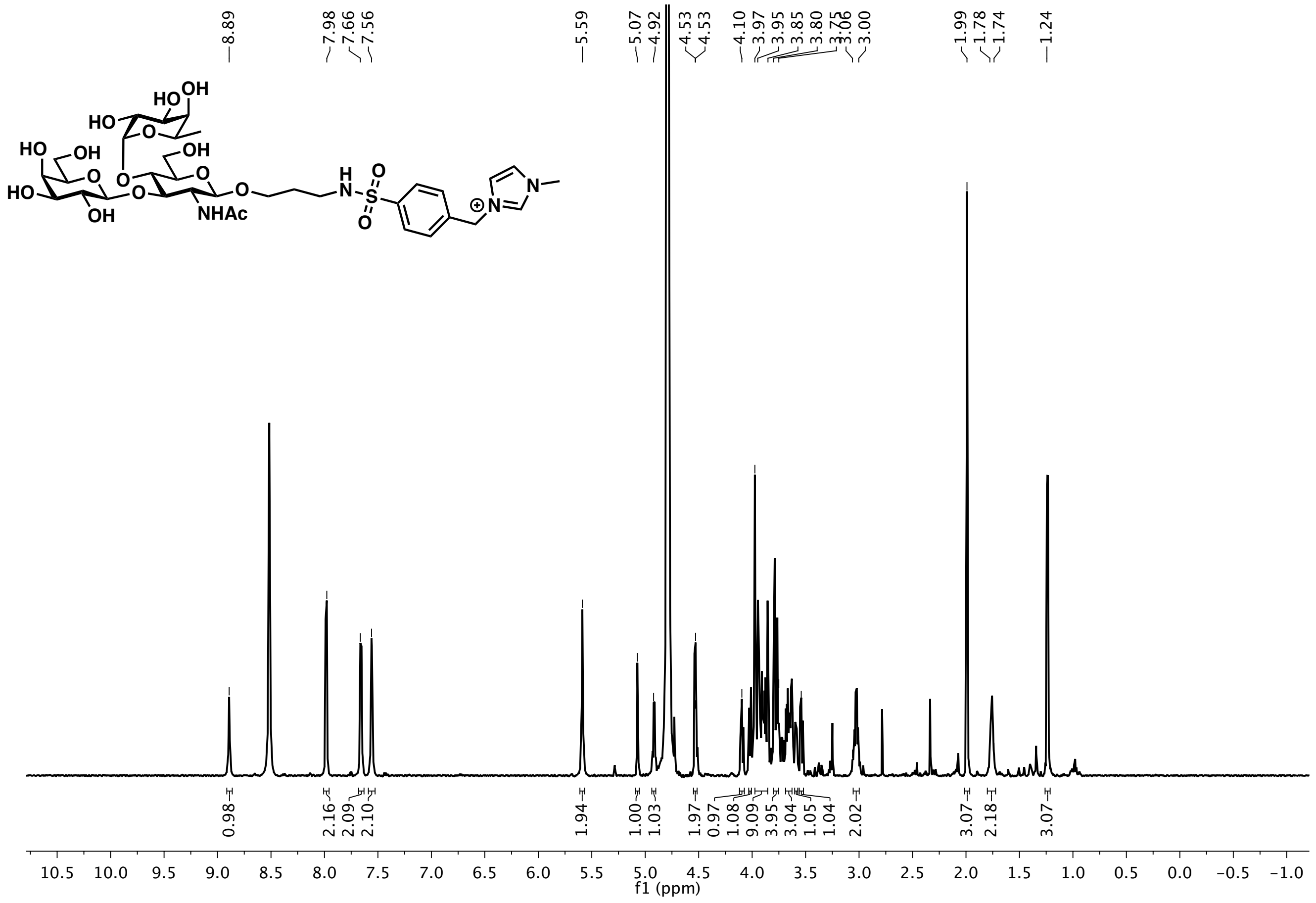


## COSY

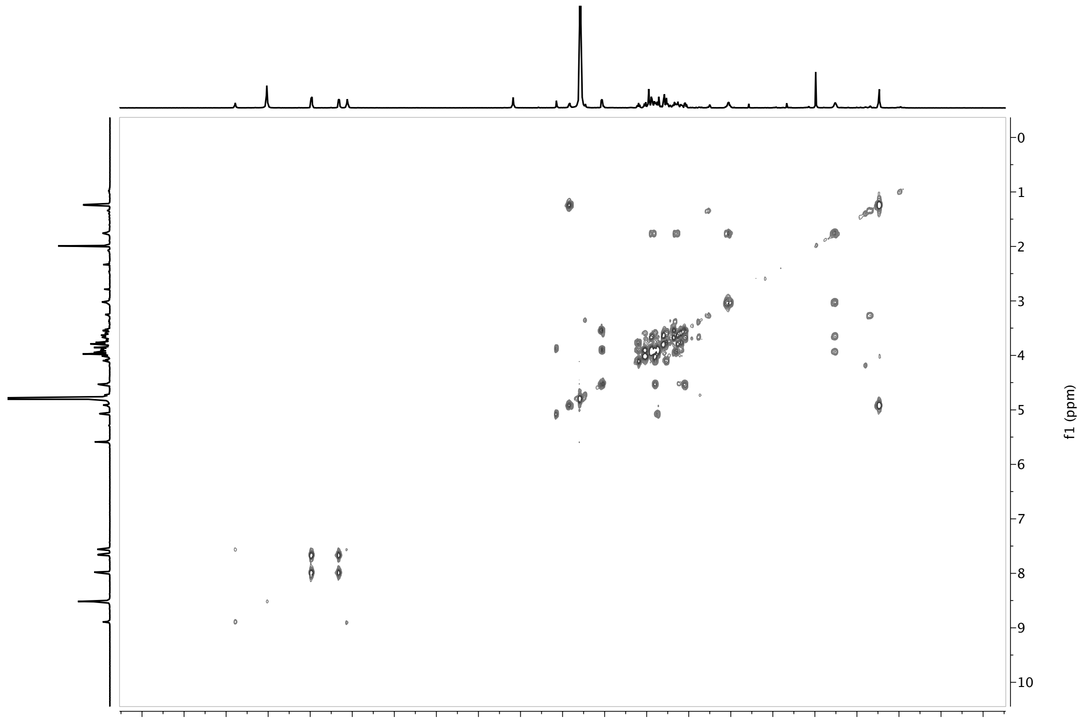

## HSQC

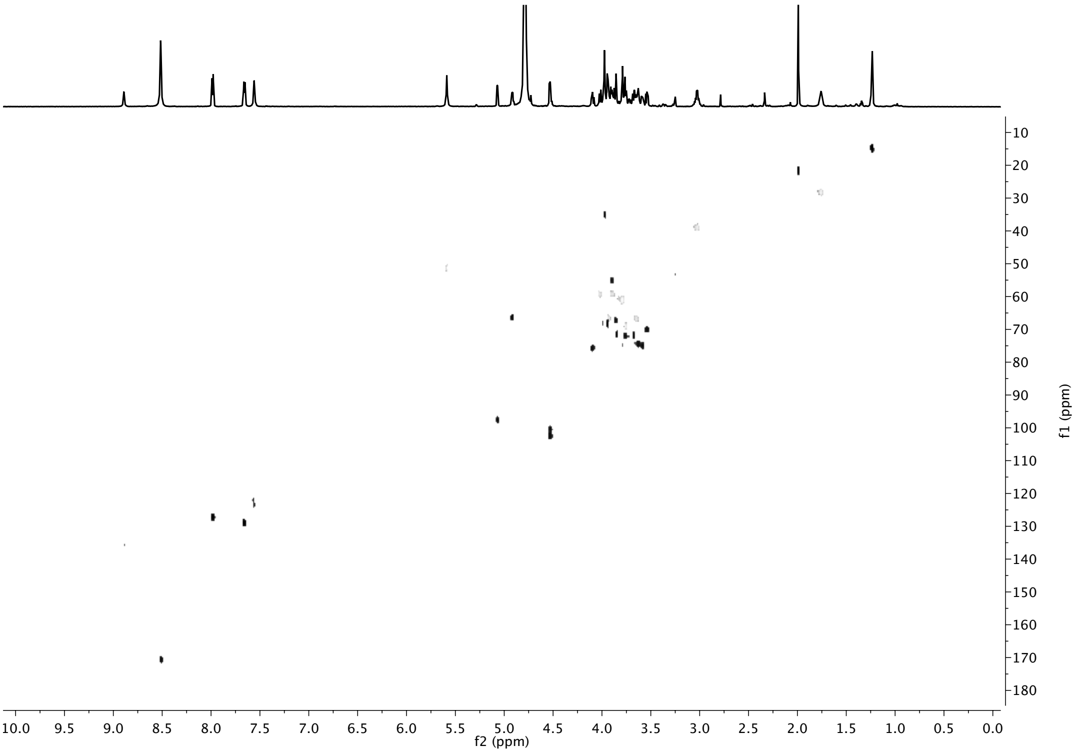


## HMBC

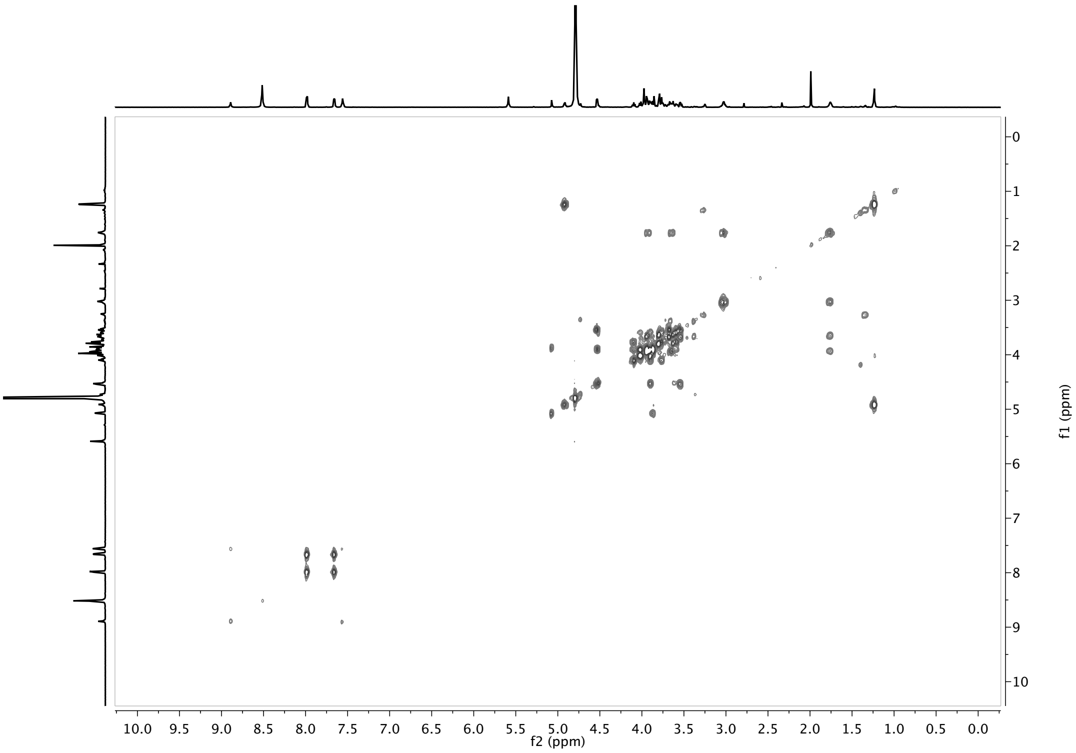

## LC-MS Traces

**One-pot *in vitro* chemo-enzymatic synthesis of ITagged human milk oligosaccharides 3, 4 and 9 from ITag-GlNAc 1.**

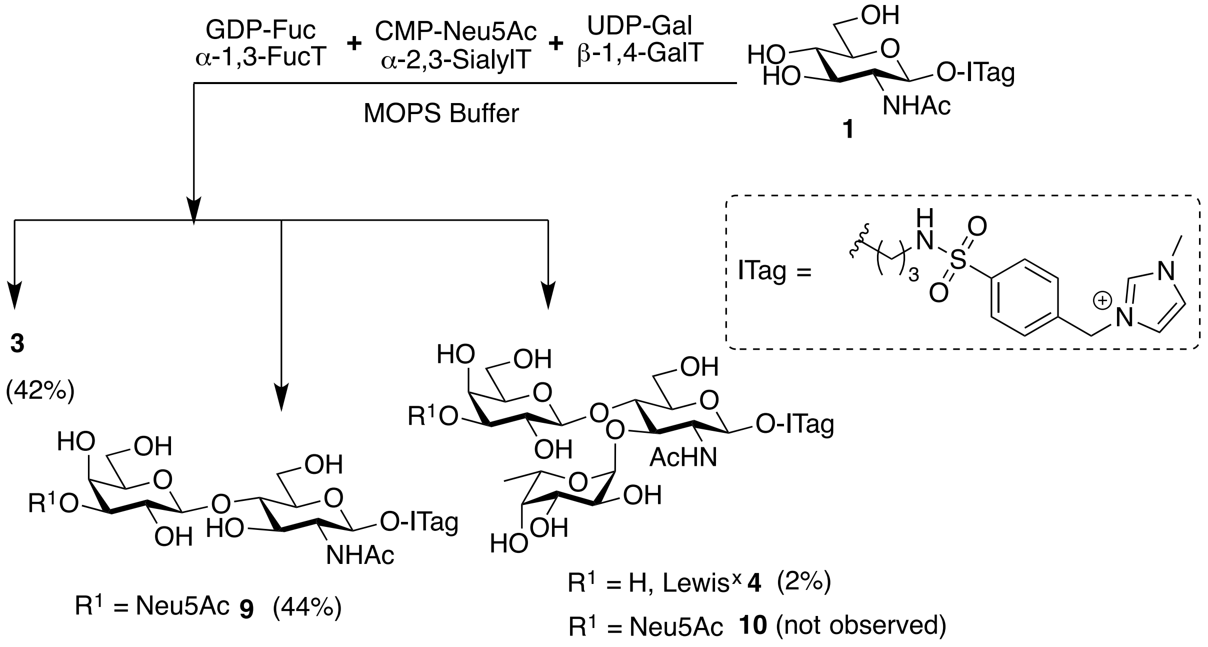

**LC-MS Chromatogram from 0-30 min.**

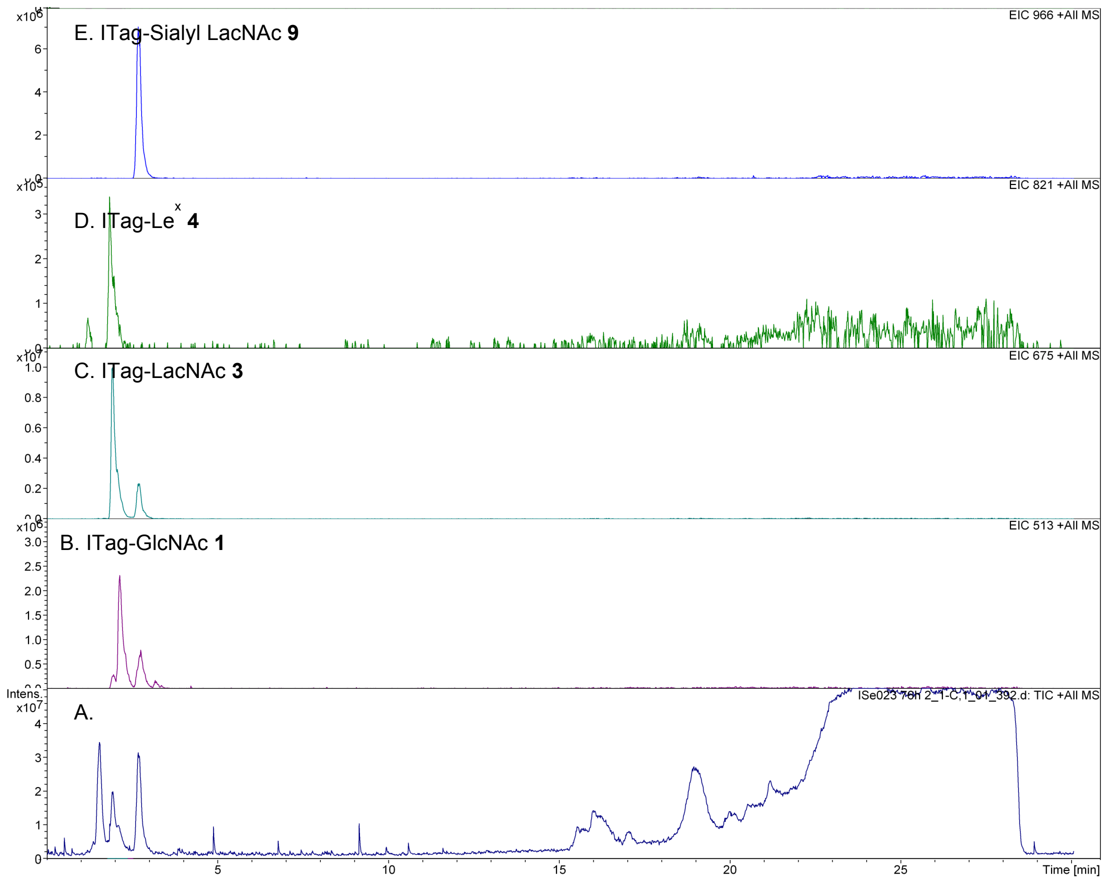


**LC-MS Chromatogram expansion from 0-5 min.**

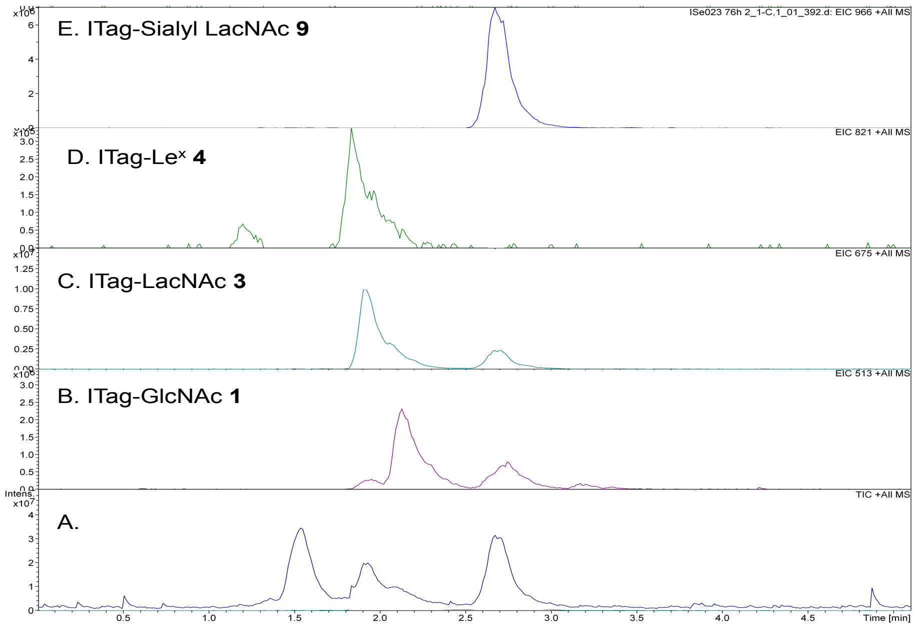


**Figure S1.** LC-MS traces (full chromatogram (*top*) and expansion (*bottom*)) for the One-pot *in vitro* chemo-enzymatic synthesis of ITagged human milk oligosaccharides **3**, **4** and **9** from ITag-GlcNAc **1** (Module C, Gradient C). A) total ion count of reaction mixture; B) extracted LC–MS traces for **1**; C) extracted LC–MS traces for **3**; D) extracted LC–MS traces for **4** and E) extracted LC–MS traces for **9**

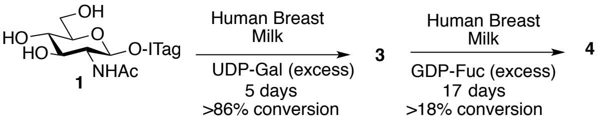


**Incubation of GlcNAc-ITag 1 and LacNAc-ITag 3 with UDP-Gal and GDP-Fuc, respectively, in HBM.**

**LC-MS Traces of untreated milk controls show that ITag-glycosides are not present in untreated HBM**

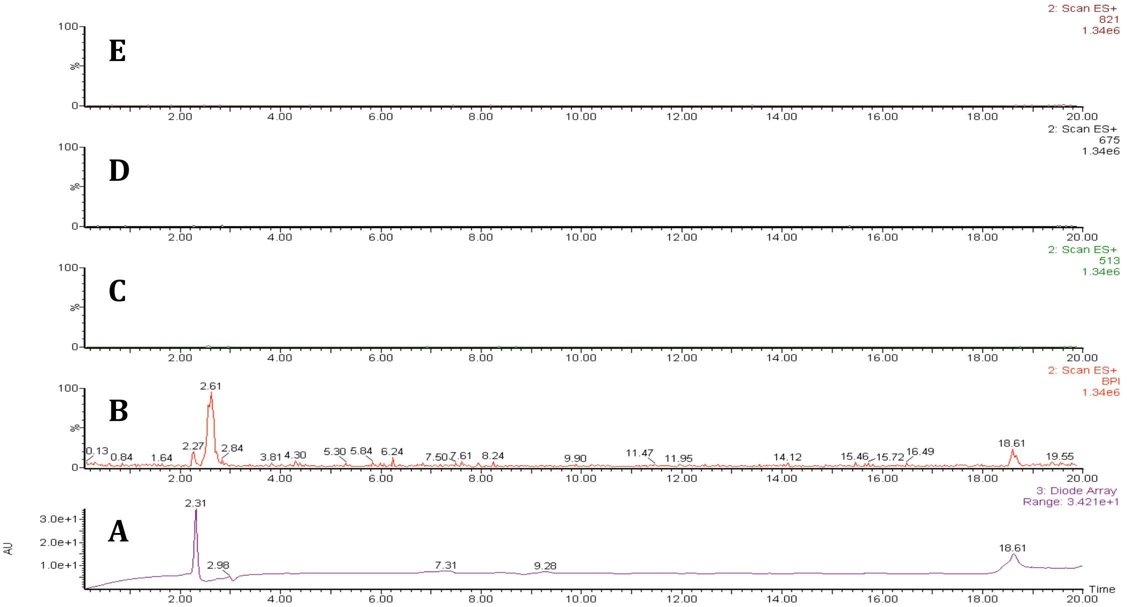


**Figure S2.** LC-MS traces of untreated HBM control (Module B, Gradient A). A) Diode array trace; B) total ion count; C) extracted LC–MS trace for **1** ($M^+$ 513); D) extracted LC–MS trace for ITag-LacNAc **3** ($M^+$ 675); E) extracted LC–MS trace for ITag-Lewis$^x$ **4** ($M^+$ 821).

**LC-MS traces of the incubation of GlcNAc-ITag 1 in HBM in the absence of UDP-Gal, which shows no conversion to disaccharide 3 or trisaccharide 4.**

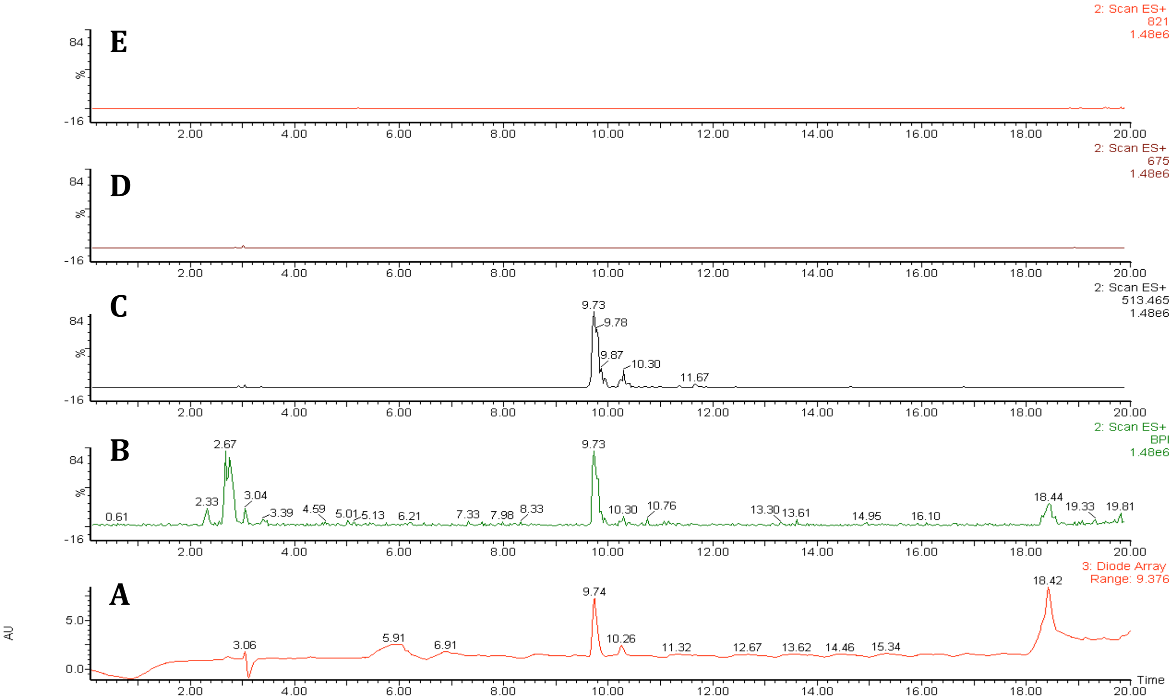


**Figure S3.** LC-MS traces of HBM incubated with **1** in the absence of UDP-Gal (Module B, Gradient A). A) Diode array trace; B) total ion count; C) extracted LC–MS trace for **1** ($M^+$ 513); D) extracted LC–MS trace for ITag-LacNAc **3** ($M^+$ 675); E) extracted LC–MS trace for ITag-Lewis$^x$ **4** ($M^+$ 821).

**LC-MS traces of the incubation of LacNAc-ITag 3 in HBM in the absence of GDP-Fuc, which shows no conversion to trisaccharide 4 or degradation to 1.**

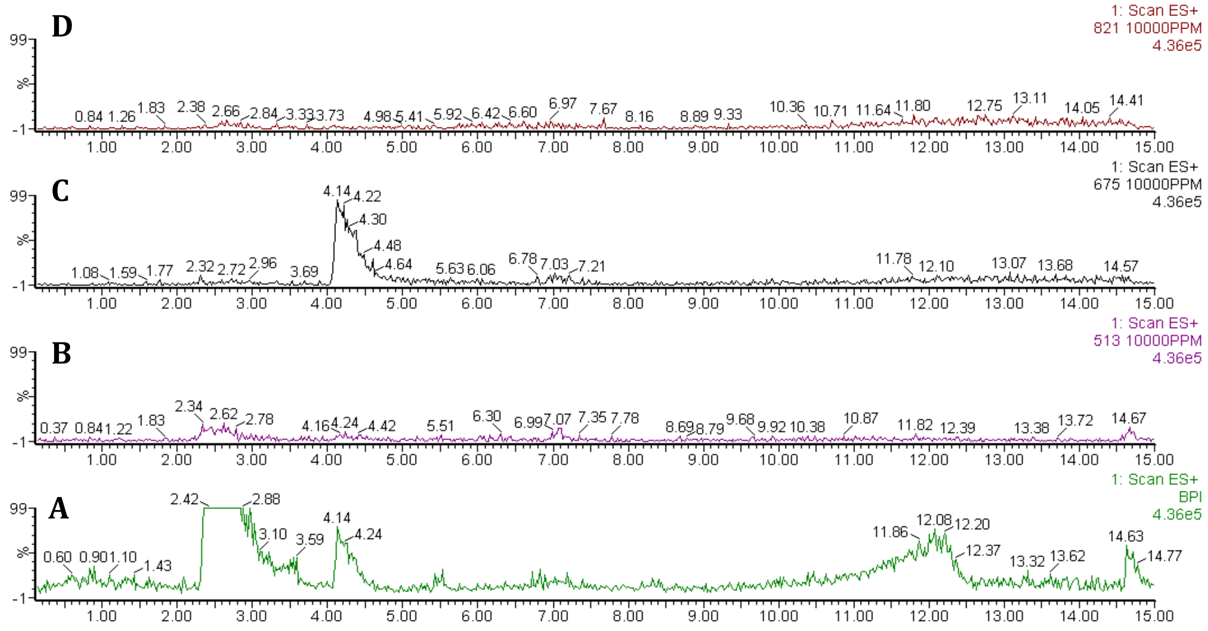


**Figure S4.** LC-MS traces of HBM incubated with **3** in the absence of GDP-Fuc (Module B, Gradient B). A) total ion count; B) extracted LC–MS trace for **1** ($M^+$ 513); C) extracted LC–MS trace for ITag-LacNAc **3** ($M^+$ 675); D) extracted LC–MS trace for ITag-Lewis$^x$ **4** ($M^+$ 821).

**LC-MS traces of the incubation of ITag-GlcNAc 1 in HBM in the presence of UDP-Gal forming ITag-LacNAc 3.**

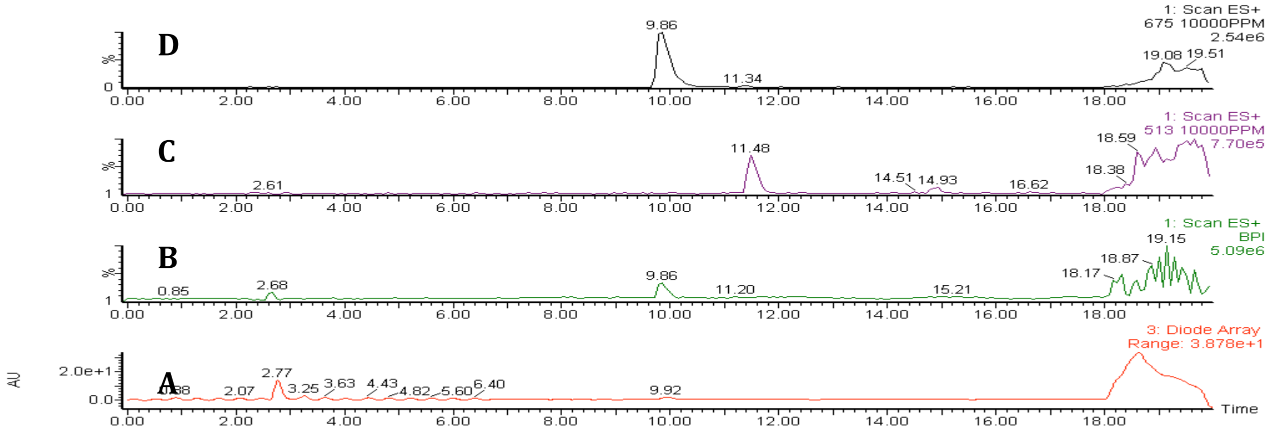


**Figure S5.** LC-MS traces of HBM incubated with **1** in the presence of UDP-Gal (Module B, Gradient A). A) Diode array trace; B) total ion count; C) extracted LC–MS trace for **1** ($M^+$ 513); D) extracted LC–MS trace for ITag-LacNAc **3** ($M^+$ 675).

**LC-MS traces of the incubation of lacto-*N*-biose in HBM in the absence of GDP-Fuc, which shows no conversion to trisaccharide 5 or degradation to 1.**

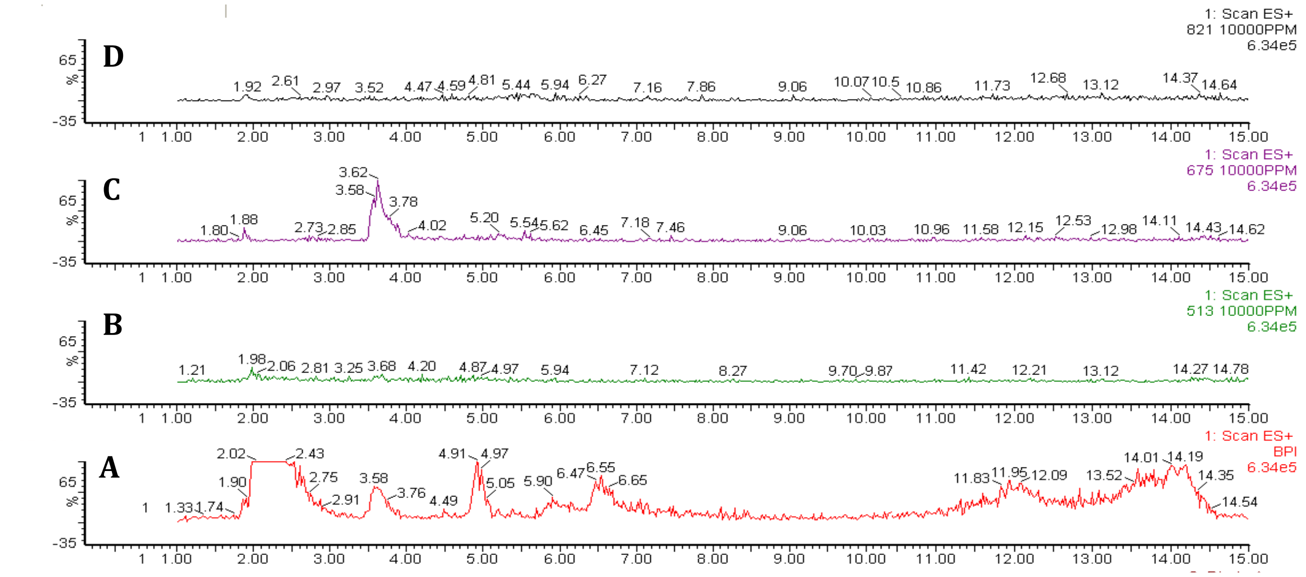

**Figure S6.** LC-MS traces of HBM incubated with **2** in the absence of GDP-Fuc (Module B, Gradient B). A) total ion count; B) extracted LC–MS trace for **1** ($M^+$ 513); C) extracted LC–MS trace for ITag-lacto-*N*-biose **2** ($M^+$ 675); D) extracted LC–MS trace for ITag-Lewis$^a$ **5** ($M^+$ 821).

**LC-MS traces of the incubation of ITag-LacNAc 3 in HBM in the presence of GDP-Fuc to produce ITag-Lewis$^x$ 4.**

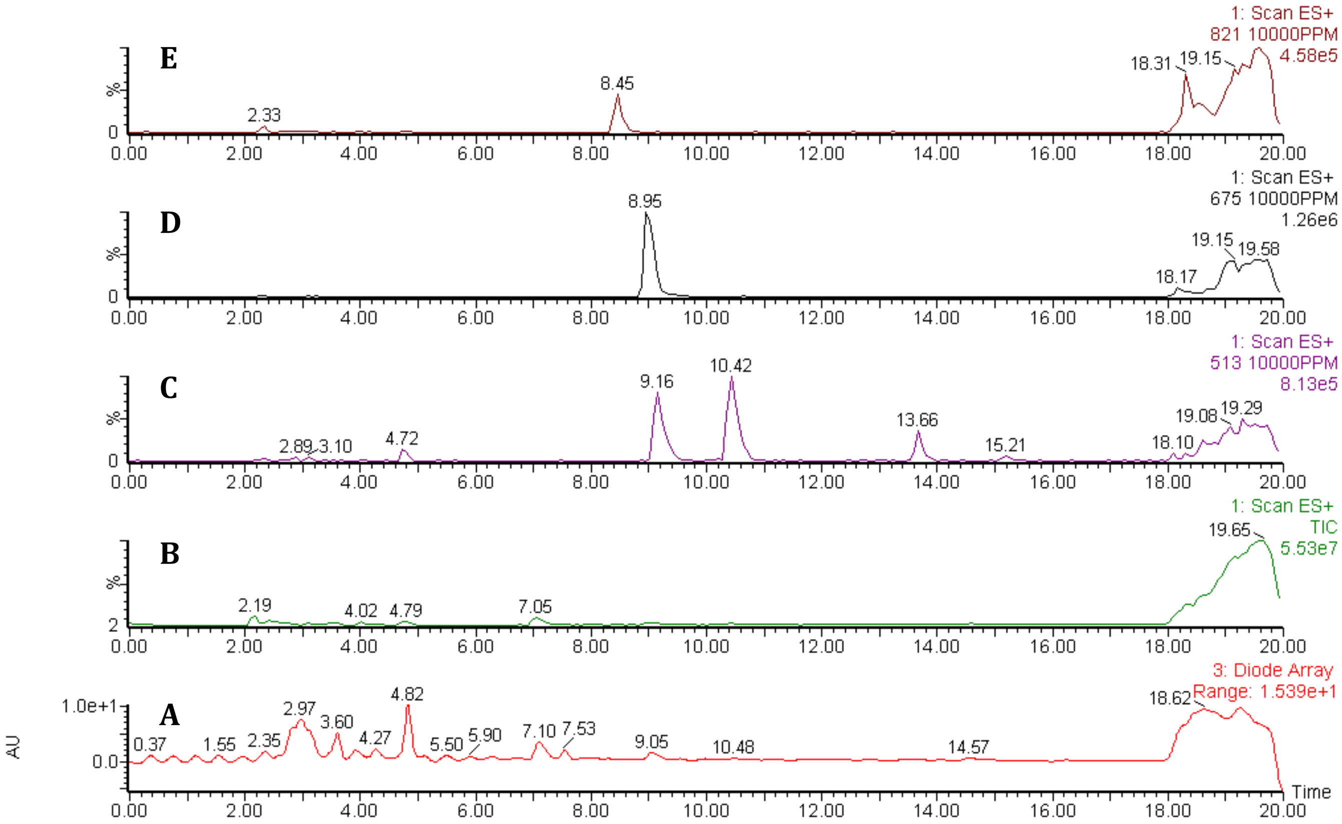

**Figure S7.** LC-MS traces of HBM incubated with **3** in the presence of GDP-Fuc (Module B, Gradient A). A) Diode array trace; B) total ion count; C) extracted LC–MS trace for **1** ($M^+$ 513); D) extracted LC–MS trace for ITag-LacNAc **3** ($M^+$ 675); E) extracted LC–MS trace for ITag-Lewis$^x$ **4** ($M^+$ 821).

**LC-MS traces of the incubation of ITag-lacto-*N*-biose 2 in HBM in the presence of GDP-Fuc to produce ITag-Lewis[a] 5.**

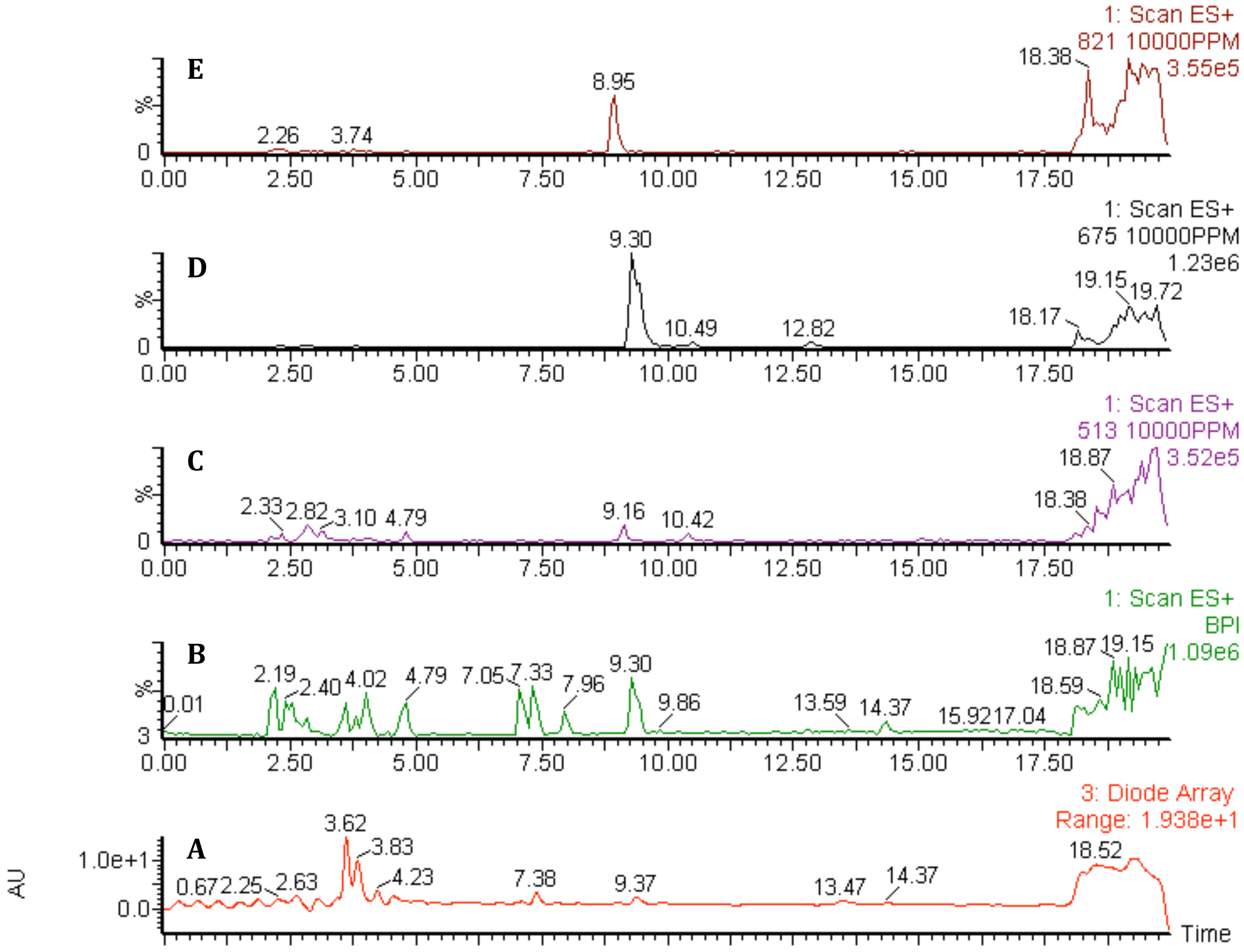


**Figure S8.** LC-MS traces of HBM incubated with **2** in the presence of GDP-Fuc (Module A, Gradient A). A) Diode array trace; B) total ion count; C) extracted LC–MS trace for **1** ($M^+$ 513); D) extracted LC–MS trace for ITag-LacNAc **3** ($M^+$ 675); E) extracted LC–MS trace for ITag-Lewis[a] **5** ($M^+$ 821).

**Reaction monitoring in human milk for:**

## Lewis$^x$

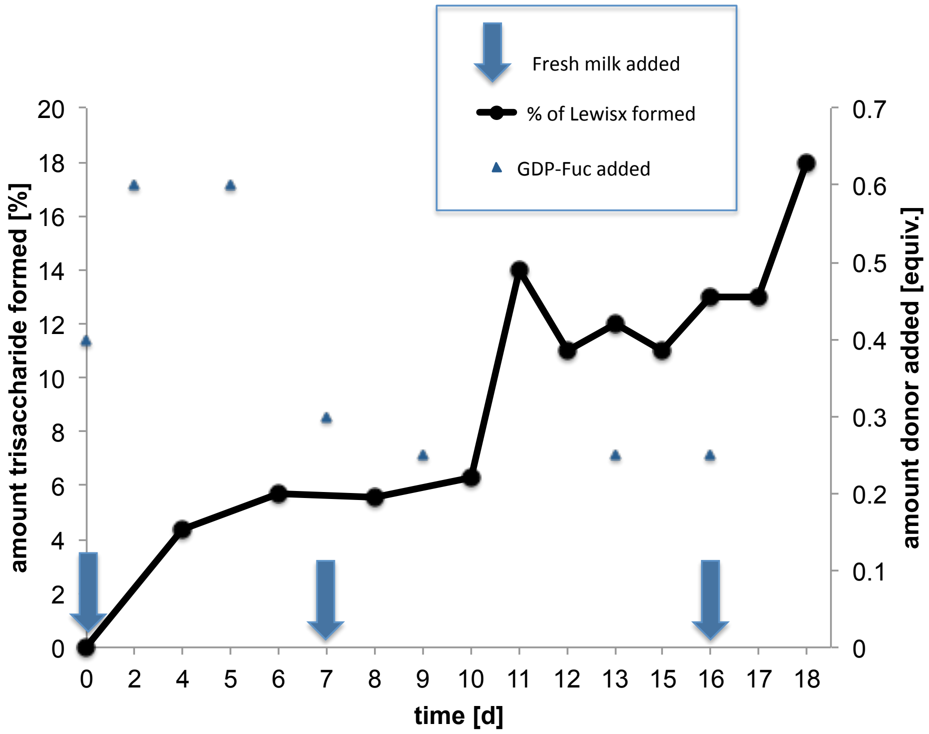


## Lewis$^a$

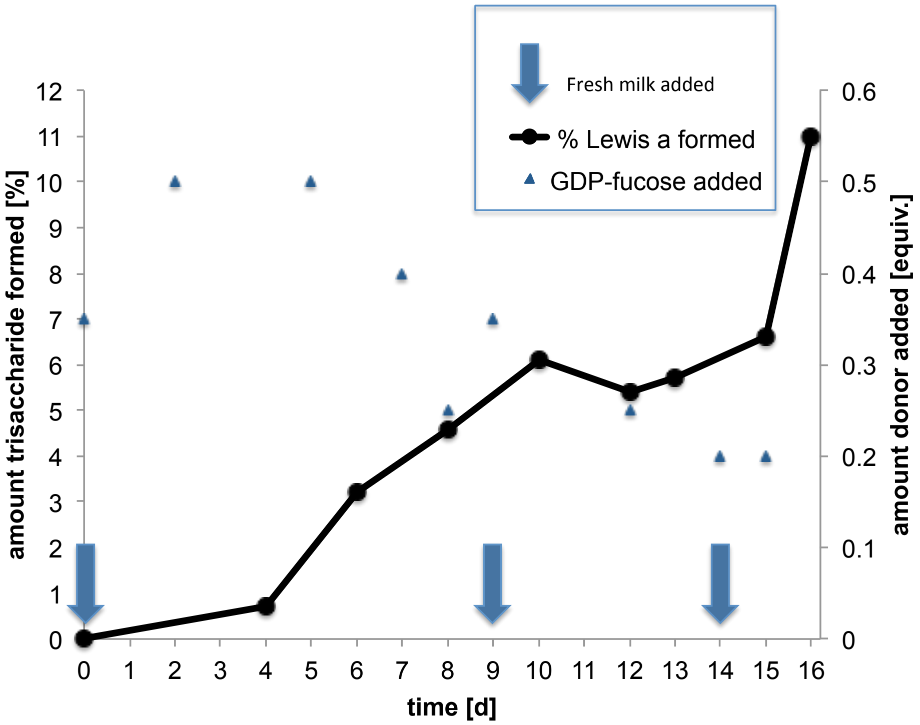


**Figure S9.** Graph showing % of trisaccharide form (Lewis$^x$ (top) and Lewis$^a$ (bottom)) as detected by LC-MS and amount of GDP-Fucose added (in equiv.) over the incubation period (days). Blue arrows indicate when fresh milk was added to the mixture.

## References


[1] M. C. Galan, A. T. Tran, K. Bromfield, S. Rabbani, B. Ernst, *Org Biomol Chem* **2012**, *10*, 7091-7097.
